# Electron scattering at a potential temporal step discontinuity

**DOI:** 10.1038/s41598-024-56168-1

**Published:** 2024-03-06

**Authors:** Furkan Ok, Amir Bahrami, Christophe Caloz

**Affiliations:** Department of Electrical Engineering, KU Leuven, Leuven, 3000 Belgium

**Keywords:** Quantum optics, Quantum physics, Matter waves and particle beams, Quantum mechanics

## Abstract

We solve the problem of electron scattering at a potential temporal step discontinuity. For this purpose, instead of the Schrödinger equation, we use the Dirac equation, for access to back-scattering and relativistic solutions. We show that back-scattering, which is associated with gauge symmetry breaking, requires a vector potential, whereas a scalar potential induces only Aharonov–Bohm type energy transitions. We derive the scattering probabilities, which are found to be of later-forward and later-backward nature, with the later-backward wave being a relativistic effect, and compare the results with those for the spatial step and classical electromagnetic counterparts of the problem. Given the unrealizability of an infinitely sharp temporal discontinuity—which is of the same nature as its spatial counterpart!—we also provide solutions for a smooth potential step and demonstrate that the same physics as for the infinitely sharp case is obtained when the duration of the potential transition is sufficiently smaller than the de Broglie period of the electron (or deeply sub-period).

## Introduction

Electron scattering at a potential *spatial* step is a canonical problem that is treated in the introductory section of most textbooks on quantum mechanics^1–6^ and that underpins uncountable phenomena (e.g., quantum reflection, transmission and interference, quantum tunneling, quantum wells and scattering resonances, quantum coherent transport) and applications (e.g., p–n junction diodes, transistors, semiconductor lasers, and detectors, scanning tunneling microscopy, quantum computing, particle accelerators). The problem is typically addressed by resolving the Schrödinger equation^7^ for non-relativistic particles, but requires promotion to the Klein–Gordon equation^8,9^ or to the Dirac equation^10^ for relativistic particles, of spin 0 or 1/2, respectively.

The problem of electron scattering at a potential *temporal* step is arguably, from space–time duality, as fundamental as that of the spatial step. A number of works on quantum phenomena occurring at step discontinuities have been reported in the literature^11–23^, but the specific problem of electron scattering at a potential temporal step has surprisingly not been explicitly resolved yet. Such a gap needs to be filled. This is all the more obvious when considering the promising opportunities of transposing to the quantum realm recent concepts developed in the booming field of *classical* electromagnetic modulation-based time-varying^24–27^ and space–time varying^28–32^ metamaterials^29,33–35^, which have already led to a wealth of novel effects and applications, including the inverse prism^36^, linear-time invariance bound breaking^37^, temporal aiming^38^, extreme energy transformation^39^, temporal antireflection coating^40^, temporal polarization conversion^41^, temporal analog computing^42,43^, static-to-dynamic field conversion^44^, temporal Faraday rotation^45–47^, arbitrary transfer function emulation^48^, optimization-free filter and matched-filter^49^, broadband parametric amplification^50,51^, wave deflection and shifted refocusing^52^ and nonreciprocity and optical isolation^53–57^.

We present here an exact and comprehensive resolution of the problem of electron scattering at a potential temporal step discontinuity. We first show that the Schrödinger equation cannot account for electron back-scattering for that problem and therefore decide to resort to the (more general) Dirac equation. We then demonstrate that a *scalar* potential temporal step does not produce back-scattering, whereas a *vector* potential temporal step does (see Supplementary Sect. 1), and explain this fact in terms of related gauge symmetry and symmetry breaking. We next derive formulas for the scattering coefficients, probabilities, and energy transitions of the electronic wave. Finally, we demonstrate that the corresponding scattering is a relativistic effect. Throughout the report, we systematically compare the problem with its spatial counterpart, and also point out some similarities and differences with corresponding electromagnetic problems^33–35^. Finally, we also provide solutions for a smooth step potential and investigate the related physics versus the transition duration of the step with respect to the de Broglie period of the electron.

## Spatial and temporal sharp potential step discontinuities

**Figure 1:**
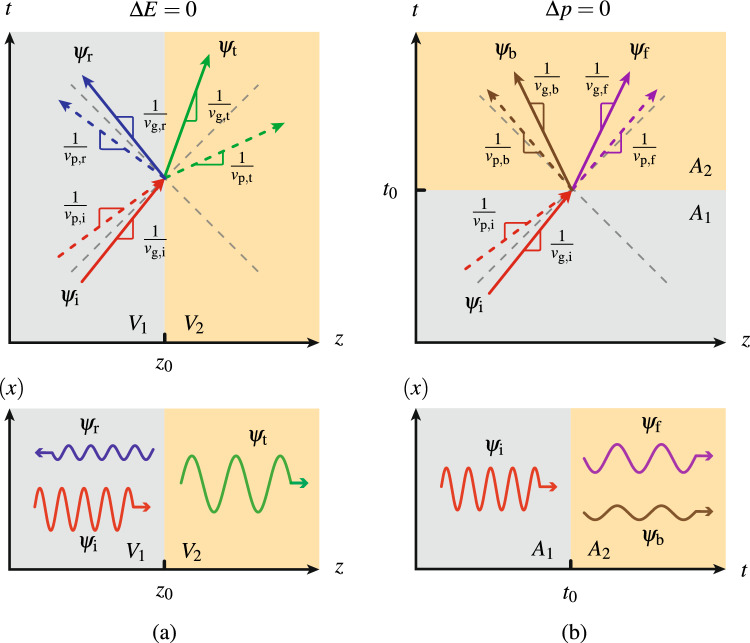
Electron scattering at a potential (**a**) spatial and (**b**) temporal step discontinuity, in spacetime (top panels) and space/time-transverse coordinates (bottom panels). The subscripts i, r, t, b, and f stand for incident, reflected, transmitted, later-backward, and later-forward, while the subscripts p and g stand for phase and group (velocity), respectively.

Figure 1 represents the problem of electron scattering at a potential step discontinuity, with the discontinuity being spatial in Fig. 1a and temporal in Fig. 1b. The latter is the problem at hand in the report while the former is considered as its dual reference. In both cases, the changing parameter is a component of the four-vector potential $A^\mu =(V,\varvec{A})$ and we shall later see why, as indicated in the figure, *V* and $\varvec{A}$ are the most relevant components for the spatial and temporal cases, respectively.

As known from textbooks, the scattered electronic waves in the spatial problem (Fig. 1a) are reflected and transmitted waves, with conserved energy ($\Delta E=0$) and transformed momentum ($\Delta {p}\ne {0}$), as in classical electromagnetics. We shall show that scattering is quite different in the counterpart temporal problem (Fig. 1b). First, the scattered electronic waves are generally *later-backward* and *later-forward* waves, as opposed to reflected and transmitted waves, where the term *later* means “after the temporal discontinuity”, contrasting with the term *earlier*, which refers to the wave launched before the temporal discontinuity, while the terms *forward* and *backward* denote corresponding propagation directions in space^33^. Second, it is now the momentum that is conserved ($\Delta p=0$), while the energy is transformed ($\Delta E\ne {0}$). These two aspects parallel the situation that prevails in classical electromagnetics^24,34^, but with a number of differences, such as the fact the phase and group velocities are generally distinct, as represented in the figure, and the fact that the later-backward wave exists only in the relativistic regime, whereas it is always present in classical electromagnetics. Throughout the paper, we shall restrict our attention to the 1+1-dimensional case, with one dimension of space (*z*) and the dimension of time (*t*). Moreover, we shall use natural units ($\hbar =c=1$) and the Minkowski metric $\eta ^{\mu \nu }=\textrm{diag}(1,-1,-1,-1)$ throughout the report.

## Choice of an appropriate equation

### Limitations of the Schrödinger equation

One may first be tempted to address the problem of the sharp temporal potential step (Fig. 1b) with the Schrödinger equation, as typically done for the sharp spatial potential step (Fig. 1a) in the non-relativistic regime. The Schrödinger equation reads $i\partial _t\psi =-{\nabla }^2\psi /(2m)$^1–6^, where *m* is the mass of the particle, which we shall consider from now on as being the electron. This equation has unpaired spatial and temporal derivatives, with the former (${\nabla }^2$) being of the second order and the latter ($\partial _t$) of the first order.

In the case of the sharp spatial step, where one typically assumes the monochromatic ansatz $\psi \propto \text {e}^{-iEt}$, the Schrödinger equation reduces to $\psi =-{\nabla }^2\psi /(2mE)$. Therefore, ${\nabla }^2\psi$ must be finite to ensure finite $\psi$, and hence $\nabla \psi$ must be continuous. In addition, $\psi$ must also be continuous, for otherwise, $\nabla \psi$ would be singular, and so would then also be $\psi \propto {\nabla }^2\psi$, and hence $\psi$. We have then the double boundary condition that both $\psi$ and ${\nabla }\psi$ must be continuous at the spatial discontinuity. Thus, the second-order derivative operator ${\nabla }^2$ provides two boundary conditions, viz., the continuity of both $\psi$ and of ${\nabla }\psi$ at the spatial discontinuity, which leads to a fully determined problem whose resolution provides the usual reflected and transmitted scattered electronic waves (Fig. 1a).

In contrast, the *first-order derivative* $\partial _t$ provides only one boundary condition in the sharp temporal potential step problem (Fig. 1b). Assuming the plane-wave ansatz $\psi \propto \text {e}^{ipz}$, the Schrödinger equation reduces to $\psi =i(2m/p^2)\partial _t\psi$ and $\partial _t\psi$ must therefore be finite to ensure finite $\psi$, which entails that $\psi$ must be continuous at the temporal discontinuity. However, this is indeed the *the only* boundary condition, due to the absence of a higher-order temporal derivative. Consequently, the Schrödinger equation does not readily include sufficient information to account for more than one scattered wave, which would involve more unknowns, specifically two unknowns in the possible case of later-forward and later-backward waves. Moreover, the Schrödinger equation is not relativistic, and would hence miss related solutions.

### Selection of the Dirac equation

The Dirac equation, which reads for the free-electron case $i\partial _t\psi =-(i\alpha ^{i}\partial _{i}-\gamma ^{0}m)\psi$, where $\psi$ is a ($4\times 1$) spinor and where $\alpha ^{i}$ and $\gamma ^{0}$ are ($4\times 4$) matrices^3,10,58,59^ (see Supplementary Sect. 2), seems to represent a safer approach for obtaining a complete solution to our temporal step problem. It also has, as the Schrödinger equation, a first-order temporal derivative order ($\partial _t$), but it involves multiple sub-equations that might together support sufficient information to account for more than one scattered wave, including relativistic ones.

Let us then try to address the problem with the Dirac equation. In order to account for the potentials in Fig. 1, we extend the free-electron Dirac equation to its minimal-coupling form^3,10,58,59^ (see Supplementary Sect. 2)

1a $$[\gamma ^{\mu }\left( i \partial _{\mu } - q A_{\mu }\right) -m] \psi =0,$$

where $\gamma ^\mu$ are the matrices (Dirac-Pauli representation)

1b $$\begin{aligned} \gamma ^{0}=\begin{pmatrix} I & 0 \\ 0 & -I \end{pmatrix} \quad \text{ and } \quad \gamma ^{i}=\begin{pmatrix} 0 & \sigma ^{i} \\ -\sigma ^{i} & 0 \end{pmatrix}, \end{aligned}$$

with *I* and $\sigma _i$ being the ($2\times {2}$) unit and Pauli matrices, respectively. Inserting the positive-energy monochromatic plane traveling wave ansatz $$\psi = \begin{pmatrix} \varphi \\ \vartheta \end{pmatrix} \textrm{e}^{-i(E t - p z)}$$ into Eq. (1) yields the general solution form (see Supplementary Sect. 3.1)

2 $$\begin{aligned} \psi = \begin{pmatrix} 1\\ 0\\ \frac{E-qV-m}{p-qA}\\ 0 \end{pmatrix} \textrm{e}^{-i(E t - p z)}, \end{aligned}$$

where $q=-e$ ($e>0$) is the charge of the electron. We make here the choice of a non-localized, continuous-wave ansatz because it is both the simplest and most appropriate regime to reveal the fundamental physics of the problem. The localized, wave-packet regime would be the next interesting regime to consider, with expected interesting novel time-delay physics, such as the quantum analog of the temporal Goos–Hänchen shift^60^.

## Scalar potential discontinuity

One may first attempt to apply the general solution (2) to the case of a pure-scalar potential, i.e., $A^\mu =(V,0)$, as typically done for the spatial step (Fig. 1a), which corresponds in the problem at hand to a temporal scalar potential step *V*(*t*), with $V(t<t_0)=V_1$ and $V(t>t_0)=V_2=V_1+\Delta {V}$, with $\Delta {V}=V_2-V_1$, where $t_0$ is the switching time. However, it may be easily verified (see Supplementary Sect. 3.2.1) that, although providing the expected energy shift (from $E_{\textrm{i}}=\sqrt{p^2+m^2}+qV_1$ to $E_{\textrm{f}}=\sqrt{p^2+m^2}+qV_2$), of potential interest for amplification applications^6^, such a potential does not produce any later-backward wave scattering! This result, which might a priori appear surprising, may be explained in terms of gauge invariance symmetry.

The (external) electric and magnetic fields, $\varvec{E}$ and $\varvec{B}$, associated with the potential modulation, are generally related to the potentials as $\varvec{E}=-{\nabla }V-\partial _t\varvec{A}$ and $\varvec{B}=\nabla \times \varvec{A}$, which are invariant under the gauge transformation^61,62^

3 $$\begin{aligned} V\rightarrow V^\prime =V-\frac{\partial {\Lambda }}{\partial {t}} \quad \text {and}\quad \varvec{A}\rightarrow \varvec{A}^\prime =\varvec{A}+{\nabla }\Lambda , \end{aligned}$$

where $\Lambda$ is an arbitrary scalar function. The sharp temporal potential step *V*(*t*) considered in the previous paragraph is equivalent to the transformation $V'=V_1+\Delta {V}\theta (t-t_0)$, where $\theta (t-t_0)$ is the Heaviside step function, and $\varvec{A}^\prime =0$, which is a particular case of the gauge transformation (3) with $V=V_1$, $-\partial _t\Lambda =\Delta V\theta (t-t_0)$, $\varvec{A}=0$ and ${\nabla }\Lambda =0$, corresponding to $\Lambda =-\Delta Vt\theta (t-t_0)$. Therefore, this sharp potential step does not involve any change in the external fields, which explains why we found that it produces no later-backward scattering. [In fact, a similar result – unchanged external fields and the consequent absence of back-scattering – is found in the case of the sharp spatial step for the potential *A*(*z*) (see Supplementary Sects. 4.2 and 3.2.3)]. The external fields are actually zero, since $\varvec{A}=0$ and ${\nabla }V={\nabla }V(t)=0$; the related energy transition due to potential without field is therefore somewhat akin to the Aharonov–Bohm effect^63^. This absence of back-scattering contrasts with the situation of the pure-scalar sharp spatial potential step *V*(*z*) (Fig. 1a), whose (reflected wave) back-scattering results from the breaking of the gauge condition (3) (see Supplementary Sect. 4.1).

## Vector potential discontinuity

We may suspect at this point that, since *V*(*t*) fails to break the gauge symmetry (3), its pure-vector potential counterpart $\varvec{A}(t)$ should break it, and hence bring about back-scattering, as the familiar (reflection) sharp spatial step *V*(*z*). That this is indeed the case is shown as follows. Assuming $\varvec{A}(t)=A(t)\varvec{\hat{z}}$, the step function reads now $A(t<t_0)=A_1$ and $A(t>t_0)=A_2=A_1+\Delta {A}$, with $\Delta {A}=A_2-A_1$. The corresponding transformation is $A^\prime =A_1+\Delta A$ $\theta (t-t_0)$ and $V'=V_1+\Delta {V}$ with $V_1=\Delta {V}=0$. Consistency with the gauge (3), given the mapping $A=A_1$, $\nabla \Lambda =\Delta \varvec{A}(t)$ or $\partial _z\Lambda =\Delta {A}\theta (t-t_0)$, $V=V_1=0$ and $-\partial _t\Lambda =0$, would now demand that $\Lambda =\Delta A\theta (t-t_0)z=\Lambda (z,t)$ along with $\Lambda \ne \Lambda (t)$. The incompatibility between the last two conditions on $\Lambda$ indicates that the transformation indeed breaks the symmetry of the gauge (3), which entails transformed external fields and which may hence lead to electron back-scattering.

We can now solve the problem of interest, for the potential, *A*(*t*), using the later-forward and later-backward ansätze corresponding to the related temporal-step classical electromagnetic solutions^24,34^ (Fig. 1b). According to Noether’s theorem^64^, for such a potential, momentum is conserved ($\Delta p=0$) due to spatial translational symmetry, viz., $p_{\textrm{i}}=p_{\textrm{f}}=p_{\textrm{b}}=p$, while broken temporal translational symmetry leads to energy transitions, which are given by the dispersion relation (see Supplementary Sects. 3.1 and 3.2.4)

4 $$\begin{aligned} E_{1,2}^{2}=\left( p-qA_{1,2}\right) ^{2}+m^{2}, \end{aligned}$$

where the subscript labels 1 and 2 refer to the earlier and later potential regions, respectively. Equation (4) leads to the energy relations

5a $$\begin{aligned} & E_{\textrm{i}}=\sqrt{(p-qA_1)^2+m^2}, \end{aligned}$$

5b $$\begin{aligned} & E_{\textrm{f}}=\sqrt{(p-qA_2)^2+m^2} \quad \text {and}\quad E_{\textrm{b}}=-E_{\textrm{f}}, \end{aligned}$$

where, assuming $E_{\textrm{f}}>0$, the apparent negative energy $E_{\textrm{b}}<0$ in the last relation simply represents propagation in the negative *z* direction ($v_{\textrm{g,b}}<0$), with positive energy ($|E_{\textrm{b}}|>0$) (see Supplementary Sect. 5).

## Dispersion and transition diagrams

**Figure 2:**
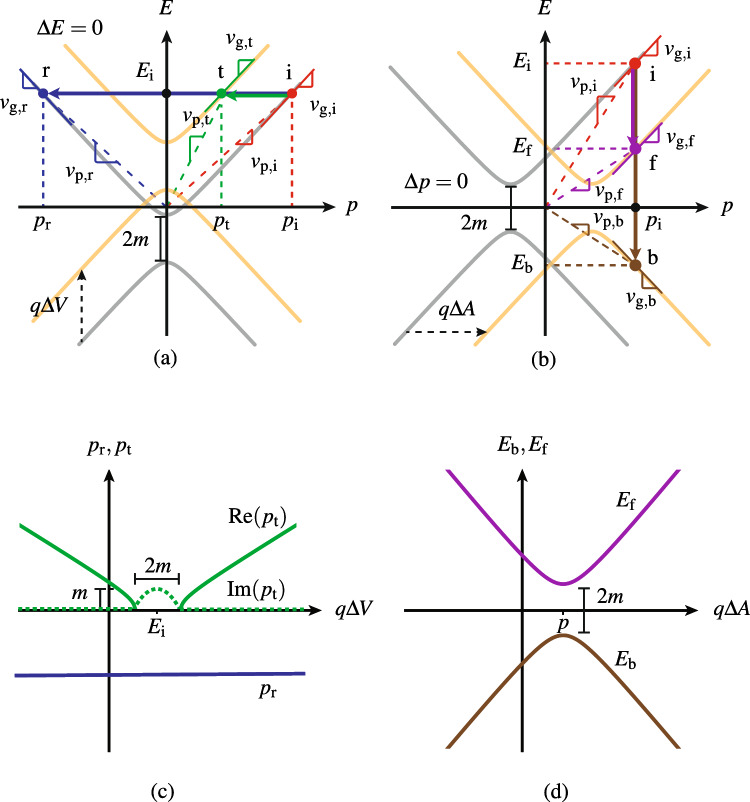
Dispersion relations and electronic transitions corresponding to Fig. 1 for (**a**) the spatial step *V*(*z*) (Fig. 1a), with (horizontal) momentum transitions, and (**b**) the temporal step *A*(*t*) (Fig. 1b), with (vertical) energy transitions, as well as corresponding (**c**) momenta and (**d**) energies versus potential steps.

Figure 2 plots the dispersion relations and electronic transitions for the two problems in Fig. 1, with Fig. 2a,b corresponding to the (reference) spatial step and temporal step problems in Fig. 1a,b, respectively, and with indications of the phase and group velocities (see Supplementary Sect. 5), corresponding to those in Fig. 1.

The spatial step (Fig. 2a) features the well-known vertical dispersion shifting with horizontal (momentum) transitions from the incident to the reflected and transmitted states (see Supplementary Sect. 3.2.2). The temporal step (Fig. 2b) [Eqs. (4) and (5)] exhibits *perfectly dual* characteristics, with horizontal dispersion shifting and vertical (energy) transitions from the earlier to the later-backward and later-forward states, whose energy levels versus $E_{\textrm{i}}$ are obtained by solving Eq. (5a) for *p* and inserting the result into Eqs. (5b), which yields

6 $$\begin{aligned} E_{\textrm{f}}=-E_{\textrm{b}}=\sqrt{\left( \sqrt{E_{\textrm{i}}^2-m^2}-q\Delta {A}\right) ^2+m^2}. \end{aligned}$$

Note that the orthogonal dispersion shifting in electronic scattering in Fig. 2 is a feature that does not exist in the classical electromagnetic counterparts of these problems, which rather involve (refractive index) dispersion curves that are rotated with respect to each other and that do not differ between the space and time cases^34^ (see Supplementary Sect. 6).

## Scattering coefficients

Upon the basis of the energy relations (5), the scattering amplitudes and probabilities may be easily found by inserting the expression for the vector potential step function *A*(*t*) into the general solution form (2) and enforcing the continuity condition $\left. \psi _{1}\right| _{t=t_0}=\left. \psi _{2}\right| _{t=t_0}$. The resulting later-backward and later-forward amplitude coefficients are (see Supplementary Sect. 3.2.4)

7a $$\begin{aligned} b = \frac{\Gamma _{\textrm{t}}-1}{2 \Gamma _{\textrm{t}}} \quad \text{ and } \quad f = \frac{1+\Gamma _{\textrm{t}}}{2 \Gamma _{\textrm{t}}}, \end{aligned}$$

corresponding to the probabilities

7b $$\begin{aligned} B = \left| b\right| ^2 \frac{2\Gamma _{\textrm{t}}^2}{1+\Gamma _{\textrm{t}}^2} \quad \text{ and } \quad F = \left| f\right| ^2 \frac{2\Gamma _{\textrm{t}}^2}{1+\Gamma _{\textrm{t}}^2}, \end{aligned}$$

where

7c $$\begin{aligned} \Gamma _{\textrm{t}} = \frac{\sqrt{\left( \sqrt{E_{\textrm{i}}^2-m^2}-(qA_2-qA_1)\right) ^2+m^2}}{\left( \sqrt{E_{\textrm{i}}^2-m^2}-(qA_2-qA_1)\right) \left( \dfrac{E_{\textrm{i}}-m}{\sqrt{E_{\textrm{i}}^2-m^2}}\right) +m}. \end{aligned}$$

Interestingly, the amplitude coefficients in Eq. (7a) are formally identical to those for classical electromagnetic scattering at a refractive index temporal step discontinuity^24,34^, with the parameter $\Gamma _{\textrm{t}}$ in Eq. (7c) replacing the refractive index contrast $N=n_2/n_1$ (see Supplementary Sect. 6).

**Figure 3:**
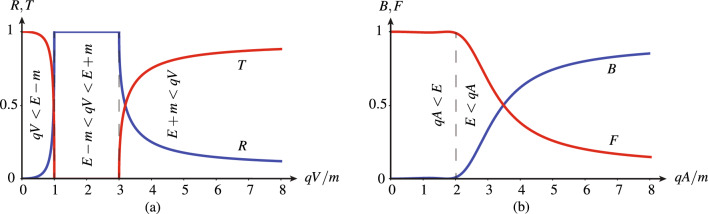
Electron scattering probabilities versus potential strength corresponding to Fig. 1 for (**a**) reflection and transmission at the spatial step *V*(*z*) (Fig. 1a) with $(V_1,V_2)=(0,V)$ and (**b**) later-backward and later-forward propagation at the temporal step *A*(*t*) (Fig. 1b) with $(A_1,A_2)=(0,A)$, for the (incident) energy to rest mass ratio $E/m=2$.

Figure 3 plots the electron scattering probabilities versus potential strength for the two problems in Fig. 1, with Fig. 3a,b corresponding to the (reference) spatial step and temporal step problems in Fig. 1a,b, respectively. The probabilities for the spatial step (Fig. 3a), also computed here from the Dirac equation (see Supplementary Sect. 3.2.2), show the well-known Klein paradox^58,65^, corresponding to the transmission gap in the range $qV=[E-m,E+m]$ and increasing transmission with increasing potential beyond the gap. In contrast, the probabilities for the temporal step (Fig. 3b) do not exhibit such a gap; they follow a monotonic trend of exchange from forward propagation at low potentials to backward propagation at high potentials. These observations interestingly suggest that a shifted Klein gap may be expected in the case of a space–time (traveling) step. The asymptotic response at high potentials ($qV/m,qA/m\gtrsim {5}$) is another fundamental difference: while the temporal step is mostly “reflective” (backward-wave) there, the spatial discontinuity is mostly transmissive, as a result of the double reflection-transmission crossing due to the Klein effect. Otherwise, the temporal step supports quasi-total forward transmission up to energies ($qA/m\approx {2}$) more than twice the cutoff of the quasi-total transmission in the spatial case ($qV/m<1$) and a forward-backward crossing point ($qA/m\approx {3.4}$) almost identical to the transmission-reflection crossing point in the spatial case ($qV/m\approx {3.2}$); these two observations correspond to trends that are generally valid when the (incident) energy is sufficient to produce a transition to the backward state, as understandable from the dispersion diagram in Fig. 2b.

## Smooth temporal potential step

The infinitely sharp temporal potential step discussed so far is a canonical structure, but it is not practically realizable, given its instantaneous transition from $A_1$ to $A_2$ and corresponding electric field singularity [$\varvec{E}=-\partial \varvec{A}/\partial {t}=-\delta (t-t_0)$]. Note that the same unrealizability issue occurs in the infinitely sharp spatial (scalar) potential discontinuity, given the dual contiguous transition from $V_1$ to $V_2$ and corresponding electric field singularity [$\varvec{E}=-\nabla {V}=-\delta (z-z_0)$]. What really matters then is to determine whether the interesting physics predicted for the infinitely sharp (unphysical) discontinuity survives as its transition is replaced by a smooth one.

**Figure 4:**
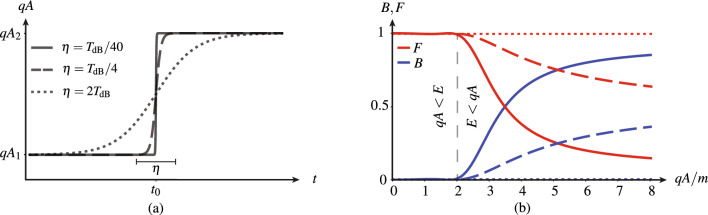
Alternative problem of a smooth temporal potential step for three different transition durations in terms of the de Broglie period, $T_{\textrm{dB}}$, for an electron with energy to rest mass ratio of $E/m=2$, as in Fig. 3. (**a**) Hyperbolic tangent function [Eq. (8)] of the related transition, between the potentials $A_1$ and $A_2$, over the time $\eta$. (**b**) Corresponding later-backward and later-forward probabilities, *B* and *F*.

For this purpose, we choose a smooth-transition potential corresponding to the hyperbolic tangent function

8 $$\begin{aligned} \varvec{A}(t) = \left[ A_1 + \frac{A_2-A_1}{2} \left( 1 + \tanh {\frac{t-t_0}{\eta }}\right) \right] \varvec{\hat{z}}, \end{aligned}$$

where the $\eta$ parameter is proportional to the transition time and whose exact Dirac solution is derived in Supplementary Sect. 7. The corresponding results are provided in Fig. 4, with Fig. 4a plotting the hyperbolic-tangent potential and Fig. 4b plotting the scattering probabilities for three representative transition times in terms of the “de Broglie period” of the electron, $T_{\textrm{dB}}$. In the sharpest case, $\eta =T_{\textrm{dB}}/40$, the scattering probabilities are indistinguishable from those for the infinitely sharp discontinuity in Fig. 3b, because the transition is *deeply sub-period*, the temporal dual regime of deep sub-wavelength. At $\eta =T_{\textrm{dB}}/4$, which may be considered as the temporal dual of the (spatial) *Fabry-Pérot condition*, the back-scattering level is less than half of that in the former case. Finally, in the smoothest case, $\eta =2T_{\textrm{dB}}$, the transition has become so slow with respect to the period, that the electron does not “see” it anymore, which results in zero back-scattering.

The deeply sub-period regime ($\eta <T_{\textrm{dB}}/10$) can unfortunately not readily be attained in current technologies, because of the extremely short de Broglie period, $T_{\textrm{dB}}=h/E=h/(2mc^2)\sim {4}\times {10}^{-21}$ s, but it might be reachable soon, given recent spectacular progress in attosecond lasers. Moreover, the present investigation can be easily extended to Dirac-type materials, such as graphene, whose de Broglie period is much smaller (e.g., $T_{\textrm{dB}}^{\textrm{graphene}} = (3a/2)/v_\text {F}\approx {3.7}\times {10}^{-16}$ s, where $a=0.246$ nm is the lattice constant and $v_\text {F}\approx {10}^{6}$ m/s is the Fermi velocity).

## Discussion

### Relativistic nature of back-scattering

The Dirac solutions in Eq. (7) and Fig. 3b confirm the validity of the later-forward wave and later-backward wave ansatz for the scattering potential $\varvec{A}=A(t)\varvec{\hat{z}}$. In the non-relativistic regime, where $\Gamma _\text {t}=1$ (see Supplementary Sect. 8), these solutions reduce to $b=0$ and $f=1$, actually corresponding to the purely later-forward solution of the Schrödinger equation. This fact reveals that later-backward scattering is a *relativistic effect*. Note that the temporal step problem, which may be seen as the infinite-velocity limit of a superluminal space–time modulation medium^34^, does not seem to be relativistic per se. Indeed, the corresponding Lorentz factor is $\gamma =1/\sqrt{1-(v_{\textrm{f}}/c)^2}$ with $v_{\textrm{f}}=c^2/v_{\textrm{m}}$, where $v_{\textrm{f}}$ is the velocity of the (instantaneous) frame and $v_{\textrm{m}}$ is the velocity of the modulation^66^, so that $\gamma {\mathop {=}\limits ^{v_{\textrm{m}}\rightarrow \infty }}1$ (no boost); it is really the speed of the electron (*v*) (not that of the modulation ($v_{\textrm{m}}$) in which it propagates) that may be relativistic in our problem. The conditional (relativistic) nature of the later-backward wave may a priori seem contradictory, given that the electromagnetic-counterpart problem unconditionally supports back-scattering^24,34^. However, considering that the particle (photon) in the latter case is inherently relativistic ($v_{\textrm{photon}}=c$), whereas it is not necessarily in the former case ($v_{\textrm{electron}}<c$), makes the finding a posteriori much less surprising.

### Experimental perspectives

#### Potential generation

It is well-known that producing a magnetic vector potential ($\varvec{A}$) to our liking might be a difficult task. This is at least the case when $\varvec{A}$ is produced from a $\varvec{B}$-field source, according to the relation $\varvec{B}=\nabla \times \varvec{A}$, as in the experiment originally proposed by Aharonov and Bohm^63^ and later realized by Tonomura et al.^67,68^, where $\varvec{A}$ is a distant effect of an enclosed $\varvec{B}$ field, theoretically requiring an infinite solenoid and producing an inconvenient curved potential^63^ or requiring a toroid with cumbersome superconductor shielding^67,68^. Fortunately, our interest here is not to produce a potential in a field-free region, as in the Aharonov–Bohm effect, but just a temporal step potential with a short transition, without any further specific restriction. This might be realized from an $\varvec{E}$-field source, according to the relation $\varvec{E}=-\partial \varvec{A}/\partial {t}$. Indeed, inserting Eq. (8) into this relation leads to the pulse function

9 $$\begin{aligned} \varvec{E}(t) = -\frac{(A_2-A_1)}{2\eta }\text {sech}^2\left( \frac{t-t_0}{\eta }\right) \varvec{\hat{z}}, \end{aligned}$$

which may be produced by an ultrashort-pulse laser^69^ to provide the desired potential step function [Eq. (8)] – collocated and aligned with the electric field. Achieving maximal back-scattering as in Fig. 3b requires, as shown in the previous section, a sub-period ($\eta <T_{\textrm{dB}}/10$) pulse, but a broader (larger $\eta$) pulse, simply generating a smoother step, may still produce some back-scattering, as shown in Fig. 4.

#### Measurement

Once the potential has been generated, as just described, electrons should be shot by an electron gun parallel to the electric field (perpendicular to the laser beam axis) and an appropriate detection procedure should be used to measure the scattering probabilities predicted by Eqs. (7b) (Fig. 3b). That detection procedure might be delicate because the physical interpretation of Dirac spinors [Eq. (2)] and related quantities is not trivial: while the operators (position, momentum, energy, spin, etc.) associated with the Schrödinger equation directly correspond to observables, those associated with the Dirac equation do not! However, the *scattering probabilities* to measure here are not problematic. Their magnitudes can be obtained by placing an electron counter in the forward region of the setup, measuring the related electron count, $N_{\textrm{F}}$, and deducing the corresponding electron count in the backward direction as $N_{\textrm{B}}=N_{\textrm{gun}}-N_{\textrm{F}}$, to obtain $F_{\textrm{meas}} = N_{\textrm{F}}/N_{\textrm{gun}}$ and $B_{\textrm{meas}} = N_{\textrm{B}}/N_{\textrm{gun}}$. Moreover, their energy levels, predicted by Eqs. (6) (Fig. 2d), can be obtained via the phase differences $\Delta \phi _{\textrm{f,b}} = -\left( E_{\textrm{i}}-E_{\textrm{f,b}}\right) t/\hbar$ measured by an electron interferometer.

### Summary and outlook

In this report, we have resolved the fundamental problem of electron scattering at a potential temporal step discontinuity, with a systematic comparison to the spatial counterpart of the problem and mention of similarities and differences with the classical electromagnetic counterparts of the two problems. The related effects described in this report might lead to a wide range of new concepts and applications in semiconductor electronics, quantum computing and information processing, and attosecond physics. A simple application would be a versatile (aligned-outputs) beam splitter, with tunable splitting ratio and splitting angle, consisting of a rotatable laser with varying intensity, where the latter controls the splitting ratio, according to Fig. 3b, and the former controls the output splitting direction.

### Supplementary Information

Supplementary Information.

## Data Availability

All data generated or analyzed during this study are included in this published article and its supplementary information files.
