# Supplementary material for: Electron scattering at a potential temporal step discontinuity
Source: Sci Rep. 2024 Mar 6;14:5559. doi: 10.1038/s41598-024-56168-1 (PMC10917777; doi:10.1038/s41598-024-56168-1)
Supplement: Supplementary file 1 — Supplementary Information. [file 41598_2024_56168_MOESM1_ESM.pdf]

Supplementary Material for “Electron Scattering at a Temporal Step Discontinuity"

Furkan Ok[1], **Amir Bahrami**[1], **and Christophe Caloz**[1,*]

[1]Department of Electrical Engineering, KU Leuven, Leuven, 3000, Belgium
[*]christophe.caloz@kuleuven.be

Contents

1 Types of Scalar and Vector Potential Spatial and Temporal Steps **2**
2 The Dirac Equation **3**
3 Solution to the Dirac Equations **5**
3.1 General Solution in the Presence of Both Scalar and Vector Potentials . . . 5
3.2 Particular Solutions in the Presence of Specific Potentials . . . 6
Scalar Potential Temporal Step, $V(t)$ • Scalar Potential Spatial Step, $V(z)$ • Vector Potential Spatial Step, $A(z)$ • Vector Potential Temporal Step, $A(t)$
4 Gauge Transformations and Symmetries **13**
4.1 Scalar Potential Spatial Step $V(z)$ . . . 13
4.2 Vector Potential Spatial Step $A(z)$ . . . 13
5 Phase and Group Velocities **14**
6 Spatial and Temporal Step Electromagnetic Problems **15**
6.1 Spatial Step Problem . . . 15
6.2 Temporal Step Problem . . . 16
7 Smooth Temporal Step **18**
7.1 Dirac Equation for a Time-Varying Vector Potential . . . 18
7.2 Solution for the Earlier Medium $(t < t_0)$ . . . 19
7.3 Solution for the Later Medium $(t > t_0)$ . . . 21
7.4 Asymptotic Forms . . . 23
Asymptotic Forms for the Earlier Medium $(t < t_0)$ • Asymptotic Forms for the Later Medium $(t > t_0)$
7.5 Weyl to Dirac Spinor Transformation . . . 24
7.6 Boundary Conditions . . . 27
7.7 Asymptotic Spinor Wavefunctions and Scattering Probabilities . . . 29
7.8 Dimensional Analysis for Time Constant τ . . . 31
8 Non-relativistic Regime **32**
8.1 Non-relativistic Limit of the Dirac Equation . . . 32
8.2 Numerical Example for the Relativistic Regime . . . 36
8.3 Spatial Scattering Coefficients in the Non-relativistic Regime . . . 36
8.4 Temporal Scattering Coefficients in the Non-relativistic Regime . . . 38
References **41**

1 Types of Scalar and Vector Potential Spatial and Temporal Steps

Figure 1 depicts the different scalar and vector potential spatial and temporal steps considered in the paper.

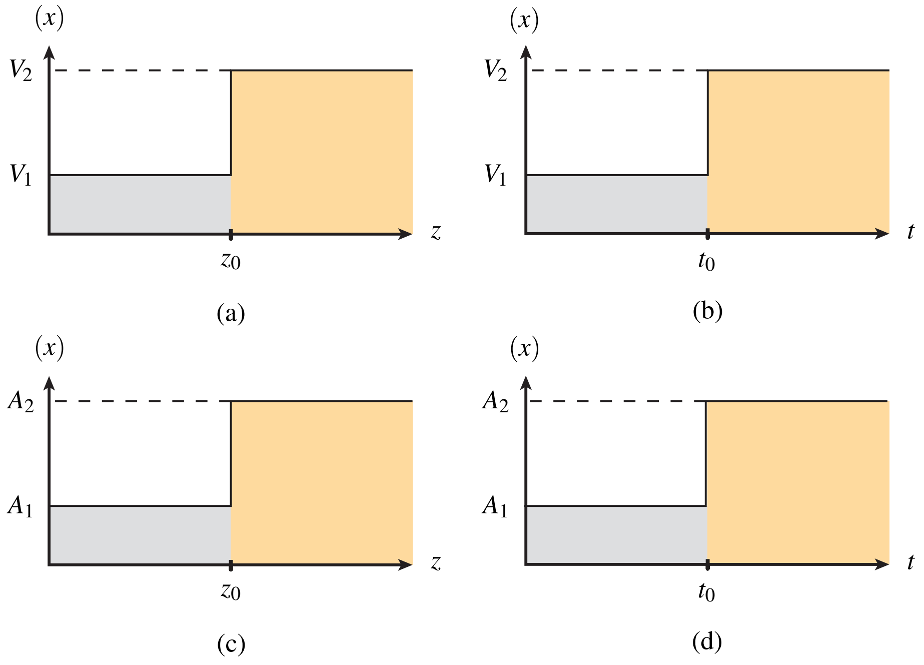

Figure 1. Types of potential steps considered. (a) Scalar potential spatial step, $V(z)$. (b) Scalar potential temporal step, $V(t)$. (c) Vector potential spatial step, $A(z)$. (d) Vector potential temporal step, $A(t)$.

2 The Dirac Equation

The Dirac equation, in natural units ($\hbar = c = 1$), reads[1,2]

$$i\frac{\partial}{\partial t}\psi = \hat{\mathscr{H}}\psi, \tag{1a}$$

where ψ is the (4×1) Dirac spinor wavefunction and $\hat{\mathscr{H}}$ is the (4×4) Hamiltonian, which is in the free (fermion) particle case

$$\hat{\mathscr{H}} = -i\alpha^i\partial_i + \gamma^0 m. \tag{1b}$$

This Hamiltonian is composed of the following elements:

- the (4×4) α^i matrices

$$\alpha^i = \begin{pmatrix} 0 & \sigma^i \\ \sigma^i & 0 \end{pmatrix}, \quad \text{with} \quad i = 1,2,3, \tag{1c}$$

whose components are the Pauli matrices

$$\sigma^1 = \begin{pmatrix} 0 & 1 \\ 1 & 0 \end{pmatrix}, \quad \sigma^2 = \begin{pmatrix} 0 & -i \\ i & 0 \end{pmatrix} \quad \text{and} \quad \sigma^3 = \begin{pmatrix} 1 & 0 \\ 0 & -1 \end{pmatrix}, \tag{1d}$$

- the four-gradient

$$\partial_\mu \equiv (\partial_0, \partial_i), \quad \text{with} \quad i = 1,2,3, \tag{1e}$$

- the (4×4) γ^0 matrix (Dirac-Pauli representation)

$$\gamma^0 = \begin{pmatrix} I & 0 \\ 0 & -I \end{pmatrix}, \quad \text{where} \quad I = \begin{pmatrix} 1 & 0 \\ 0 & 1 \end{pmatrix}, \tag{1f}$$

- the particle mass, m.

Inserting Eq. (1b) into Eq. (1a) multiplied by γ^0 yields

$$i\gamma^0\partial_0\psi = \left(-i\gamma^0\alpha^i\partial_i + \gamma^0\gamma^0 m\right)\psi. \tag{2}$$

Then combining Eqs. (1f) and (1c), which gives

$$\gamma^0\alpha^i = \begin{pmatrix} 0 & \sigma^i \\ -\sigma^i & 0 \end{pmatrix} = \gamma^i, \tag{3}$$

and noting that $\gamma^0\gamma^0 = 1$, simplifies Eq. (2) to

$$i\gamma^0\partial_0\psi = \left(-i\gamma^i\partial_i + m\right)\psi. \tag{4}$$

Finally, moving the right-hand side terms of this equation to its left-hand side provides the explicit form of the free-particle Dirac equation,

$$\left(i\gamma^\mu\partial_\mu - m\right)\psi = 0, \quad \text{with} \quad \mu = 0,1,2,3, \tag{5}$$

which assumes the Einstein summation convention, whereby the repetition of an index in a given term implies summation over that index. Note that Eq. (5) represents four scalar equations via the γ^μ matrices.

When the particle is subjected to an electromagnetic field, expressed in terms of the four-potential[3]

$$A_\mu = (A_0, -\mathbf{A}), \tag{6}$$

the Dirac equation (5) transforms according to the minimal-coupling prescription[4]

$$\partial_\mu \to \partial_\mu + iqA_\mu, \tag{7}$$

viz.,

$$\left[\gamma^{\mu}\left(i\partial_{\mu} - qA_{\mu}\right) - m\right]\psi = 0, \tag{8}$$

where q is the charge of the particle.

The Dirac equation (8) may then be solved with the general ansatz

$$\psi = \begin{pmatrix} \varphi \\ \vartheta \end{pmatrix} \mathrm{e}^{\mp ip\cdot x}, \tag{9}$$

where φ and ϑ are two-component (2×1) spinors and

$$p \cdot x = p^{\mu} x_{\mu} = \eta^{\mu\sigma} p_{\sigma} x_{\mu}, \tag{10}$$

with

$$\eta^{\mu\sigma} = \begin{pmatrix} 1 & 0 & 0 & 0 \\ 0 & -1 & 0 & 0 \\ 0 & 0 & -1 & 0 \\ 0 & 0 & 0 & -1 \end{pmatrix} \tag{11}$$

being the Minkowski metric (West Coast convention). That the ansatz (9) is correct will be verified in Sec. 3. In Eq. (10),

$$p_{\mu} = (E, -\mathbf{p}) \tag{12}$$

is the momentum four-vector and

$$x_{\mu} = (t, -\mathbf{x}) \tag{13}$$

is the position four-vector. The expression $p \cdot x = Et - \mathbf{p} \cdot \mathbf{x}$ is then identified as the phase of a plane wave, which reveals that the solution form in Eq. (9) corresponds to a plane-wave solution. We see then that two signs in that equation [Eq. (9)] correspond to positive and negative energies, which Dirac identified as corresponding to the particle (e.g., electron) and its antiparticle (e.g., positron). Assuming that we are exclusively dealing with the former type, we shall ignore negative energies and hence drop the positive sign in the spinor waveform (9). Thus, further restricting our attention to the $1+1$ dimensional case, with the spatial dimension being z, we find that the ansatz (9) reduces to the specific form

$$\psi = \begin{pmatrix} \varphi \\ \vartheta \end{pmatrix} \mathrm{e}^{-i(Et - pz)}. \tag{14}$$

3 Solution to the Dirac Equations

3.1 General Solution in the Presence of Both Scalar and Vector Potentials

Substituting the ansatz (14) into the Dirac equation (8) gives

$$\left[\gamma^{\mu}(i\partial_{\mu} - qA_{\mu}) - m\right]\begin{pmatrix}\varphi \\ \vartheta\end{pmatrix} \mathrm{e}^{-i(Et-pz)} = 0. \tag{15a}$$

Expanding this relation according to the Einstein summation convention yields

$$\left[\gamma^{0}(i\partial_{0} - qA_{0}) + \gamma^{3}(i\partial_{3} + qA_{3}) - m\right]\begin{pmatrix}\varphi \\ \vartheta\end{pmatrix} \mathrm{e}^{-i(Et-pz)} = 0, \tag{15b}$$

which becomes after applying the derivative operators to the exponential term

$$\left[\gamma^{0}(E - qA_{0}) - \gamma^{3}(p - qA_{3}) - m\right]\begin{pmatrix}\varphi \\ \vartheta\end{pmatrix} \mathrm{e}^{-i(Et-pz)} = 0. \tag{15c}$$

Inserting now the gamma matrices γ^0 and γ^3 from Eqs. (1f) and (3), respectively, into this relation and dropping the exponential function, leads to

$$\left[\begin{pmatrix}I & 0 \\ 0 & -I\end{pmatrix}(E - qA_{0}) - \begin{pmatrix}0 & \sigma^{3} \\ -\sigma^{3} & 0\end{pmatrix}(p - qA_{3}) - m\right]\begin{pmatrix}\varphi \\ \vartheta\end{pmatrix} = 0, \tag{15d}$$

which splits into the two equations

$$(E - qA_0)\varphi - (p - qA_3)\sigma^3\vartheta - m\varphi = 0 \tag{16a}$$

and

$$-(E - qA_0)\vartheta + (p - qA_3)\sigma^3\varphi - m\vartheta = 0. \tag{16b}$$

Separately solving these equations for φ yields then

$$\varphi = \frac{(p - qA_3)\sigma^3}{E - qA_0 - m}\vartheta \tag{17a}$$

and

$$\varphi = \frac{E - qA_0 + m}{(p - qA_3)\sigma^3}\vartheta. \tag{17b}$$

Equating these two relations and using the identity $(\sigma^3)^2 = I$ that follows from Eq. (1d), entails the Dirac energy-momentum or dispersion relation

$$(E - qA_0)^2 = (p - qA_3)^2 + m^2, \tag{18}$$

which reduces for $A_0 = A_3 = 0$ to Einstein's equation $E^2 = p^2 + m^2 = (\gamma m)^2$, where $\gamma = (1 - v^2)^{-1/2}$ is the Lorentz factor, with v begin the velocity of the particle.

At this point, we may select either the spin-up solution or the spin-down solution, which correspond to the spinors

$$\varphi_{\uparrow} = \begin{pmatrix}1 \\ 0\end{pmatrix} \tag{19a}$$

and

$$\varphi_{\downarrow} = \begin{pmatrix}0 \\ 1\end{pmatrix}, \tag{19b}$$

respectively. Choosing the spin-up solution and correspondingly inserting Eq. (19a) into Eq. (17a), yields

$$\begin{pmatrix}1 \\ 0\end{pmatrix} = \frac{p - qA_3}{E - qA_0 - m}\begin{pmatrix}1 & 0 \\ 0 & -1\end{pmatrix}\begin{pmatrix}\vartheta_1 \\ \vartheta_2\end{pmatrix} \tag{20a}$$

or

$$\begin{pmatrix}1\\0\end{pmatrix} = \frac{p-qA_3}{E-qA_0-m}\begin{pmatrix}\vartheta_1\\-\vartheta_2\end{pmatrix}, \tag{20b}$$

which determines the spinor as ϑ as

$$\vartheta = \begin{pmatrix}\vartheta_1\\\vartheta_2\end{pmatrix} = \begin{pmatrix}\frac{E-qA_0-m}{p-qA_3}\\0\end{pmatrix}. \tag{21}$$

Finally, substituting Eq. (19a) and Eq. (21) into (14) yields the solution form

$$\psi = \begin{pmatrix}1\\0\\\frac{E-qA_0-m}{p-qA_3}\\0\end{pmatrix}\mathrm{e}^{-i(Et-pz)}. \tag{22}$$

Note that the alternative choice of Eq. (17b) would have led to the third element of this spinor being replaced by $\frac{p-qA_3}{E-qA_0+m}$, which can easily be shown to be equal to $\frac{E-qA_0-m}{p-qA_3}$. Further note that the alternative choice of the spin-down solution [Eq. (19b)] would have led to the entries 1 and $\frac{E-qA_0-m}{p-qA_3}$ being replaced by zero and shifted to the second and fourth slots of the spinor, respectively, with the sign of the latter changed.

3.2 Particular Solutions in the Presence of Specific Potentials

We shall now apply the general solution in Eq. (22) to the different step potential introduced in Sec. 1.

3.2.1 Scalar Potential Temporal Step, $V(t)$

The scalar potential temporal step, shown in Fig. 1(b), may be written

$$A_0 = V(t) = \begin{cases} V_1 & \text{for } t < t_0, \\ V_2 & \text{for } t > t_0, \end{cases} \quad \text{and} \quad A_3 = 0. \tag{23}$$

Substituting this potential into the solution form (22) and using ansätze corresponding to the related temporal-step electromagnetic solutions[5,6], we assume the incident, or earlier ($t < t_0$), spinor wavefunction

$$\psi_1 = \begin{pmatrix}1\\0\\\frac{E_\mathrm{i}-qV_1-m}{p_\mathrm{i}}\\0\end{pmatrix}\mathrm{e}^{-iE_\mathrm{i}t}\mathrm{e}^{ip_\mathrm{i}z} \tag{24a}$$

and the later ($t > t_0$) spinor wavefunction

$$\psi_2 = f\begin{pmatrix}1\\0\\\frac{E_\mathrm{f}-qV_2-m}{p_\mathrm{f}}\\0\end{pmatrix}\mathrm{e}^{-iE_\mathrm{f}t}\mathrm{e}^{ip_\mathrm{f}z} + b\begin{pmatrix}1\\0\\\frac{E_\mathrm{b}-qV_2-m}{p_\mathrm{b}}\\0\end{pmatrix}\mathrm{e}^{-iE_\mathrm{b}t}\mathrm{e}^{ip_\mathrm{b}z}, \tag{24b}$$

whose first and second terms correspond to later-forward and later-backward waves, respectively, with corresponding amplitude coefficients f and b.

According to Noether's theorem[7], momentum is conserved ($\Delta p = 0$) due to spatial translational symmetry, viz.,

$$p_\mathrm{i} = p_\mathrm{f} = p_\mathrm{b} = p, \tag{25a}$$

whereas the breaking of temporal symmetry entails energy transformations, which are found from Eq. (18) with $A_0 = V(t)$ and $A_3 = 0$ to be

$$E_\mathrm{i} = \sqrt{p^2+m^2} + qV_1, \tag{25b}$$

$$E_{\mathrm{f}} = \sqrt{p^2+m^2}+qV_2 \tag{25c}$$

and

$$E_{\mathrm{b}} = -E_{\mathrm{f}}+2qV_2, \tag{25d}$$

where the apparent negative energy in the last relation simply represents propagation in the negative z direction, with positive energy.

According to Eq. (8), the spinor wavefunction must be continuous at the temporal discontinuity, viz.,

$$\psi_1|_{t=t_0} = \psi_2|_{t=t_0}. \tag{26}$$

Inserting Eqs. (24) into this relation yields

$$1 = f+b \tag{27a}$$

and

$$\frac{E_{\mathrm{i}}-qV_1-m}{p_{\mathrm{i}}} = f\frac{E_{\mathrm{f}}-qV_2-m}{p_{\mathrm{f}}}+b\frac{E_{\mathrm{b}}-qV_2-m}{p_{\mathrm{b}}}. \tag{27b}$$

Substituting then Eqs. (25) into Eq. (27b), and solving the system formed by the resulting equation and Eq. (27a), finally leads, after some algebraic manipulations, to

$$f = 1 \quad \text{and} \quad b = 0, \tag{28}$$

which reveals that the scalar potential temporal step, $V(t)$, does not produce any back-scattering.

3.2.2 Scalar Potential Spatial Step, $V(z)$

The scalar potential spatial step, shown in Fig. 1(a), may be written

$$A_0 = V(z) = \begin{cases} V_1 & \text{for } z<z_0, \\ V_2 & \text{for } z>z_0, \end{cases} \quad \text{and} \quad A_3 = 0. \tag{29}$$

Substituting this potential into the solution form (22) and using ansätze corresponding to the related spatial-step electromagnetic solutions[3,6], we assume the left ($z<z_0$) spinor wavefunction

$$\psi_1 = \begin{pmatrix} 1 \\ 0 \\ \frac{E_{\mathrm{i}}-qV_1-m}{p_{\mathrm{i}}} \\ 0 \end{pmatrix} \mathrm{e}^{-iE_{\mathrm{i}}t}\mathrm{e}^{ip_{\mathrm{i}}z}+r\begin{pmatrix} 1 \\ 0 \\ \frac{E_{\mathrm{r}}-qV_1-m}{p_{\mathrm{r}}} \\ 0 \end{pmatrix} \mathrm{e}^{-iE_{\mathrm{r}}t}\mathrm{e}^{ip_{\mathrm{r}}z}, \tag{30a}$$

whose first and second terms correspond to incident and reflected waves, respectively, with reflection amplitude coefficient r, and the transmitted, or right ($z>z_0$), spinor wavefunction

$$\psi_2 = t\begin{pmatrix} 1 \\ 0 \\ \frac{E_{\mathrm{t}}-qV_2-m}{p_{\mathrm{t}}} \\ 0 \end{pmatrix} \mathrm{e}^{-iE_{\mathrm{t}}t}\mathrm{e}^{ip_{\mathrm{t}}z}, \tag{30b}$$

with transmission amplitude coefficient t.

According to Noether's theorem[7], energy is conserved ($\Delta E = 0$) due to temporal translational symmetry, viz.,

$$E_{\mathrm{i}} = E_{\mathrm{r}} = E_{\mathrm{t}} = E, \tag{31a}$$

whereas the breaking of spatial symmetry implies momentum transformations, which are found from Eq. (18) with $A_0 = V(z)$ and $A_3 = 0$ to be

$$p_{\mathrm{i}} = \sqrt{(E-qV_1)^2-m^2}, \tag{31b}$$

$$p_{\mathrm{r}} = -p_{\mathrm{i}} \tag{31c}$$

and

$$p_{\mathrm{t}} = \sqrt{(E - qV_2)^2 - m^2}, \tag{31d}$$

where the apparent negative momentum in Eq. (31c) simply represents propagation in the negative z direction, with positive momentum, whose momentum versus p_{i} are obtained by solving Eq. (31b) for E and inserting the result into Eqs. (31c) and (31d), which yields

$$p_{\mathrm{t}} = \sqrt{\left(\sqrt{p_{\mathrm{i}}^2 + m^2} - q\Delta V\right)^2 - m^2}. \tag{31e}$$

According to Eq. (8), the spinor wavefunction must be continuous at the spatial discontinuity, viz.,

$$\psi_1|_{z=z_0} = \psi_2|_{z=z_0}. \tag{32}$$

Inserting Eqs. (30) in this relation yields

$$1 + r = t \tag{33a}$$

and

$$\frac{E_{\mathrm{i}} - qV_1 - m}{p_{\mathrm{i}}} + r\frac{E_{\mathrm{r}} - qV_1 - m}{p_{\mathrm{r}}} = t\frac{E_{\mathrm{t}} - qV_2 - m}{p_{\mathrm{t}}}. \tag{33b}$$

Substituting then Eqs. (31) into Eq. (33b), and solving the system formed by the resulting equation and Eq. (33a), finally leads, after some algebraic manipulations, to

$$r = \frac{1 - \Gamma_{\mathrm{s}}}{1 + \Gamma_{\mathrm{s}}} \quad \text{and} \quad t = \frac{2}{1 + \Gamma_{\mathrm{s}}}, \tag{34a}$$

where

$$\Gamma_{\mathrm{s}} = \frac{(E - qV_2 - m)p_{\mathrm{i}}}{(E - qV_1 - m)p_{\mathrm{t}}}. \tag{34b}$$

The probabilities associated with the reflected and transmitted waves may then be found from the ratios of the corresponding components of the conserved Dirac current. The Dirac conserved current is

$$j^{\mu} = \bar{\psi}\gamma^{\mu}\psi, \tag{35}$$

where $\bar{\psi}$ is the Dirac adjoint,

$$\bar{\psi} = \psi^{\dagger}\gamma^0, \tag{36}$$

with $\psi^{\dagger}$ being the Hermitian conjugate of ψ. In our problem [Fig. 1(a)], j^{μ} has only a z spatial component, and takes then the forms

$$j_1^z = \psi_1^{\dagger}\gamma^0\gamma^3\psi_1 \tag{37a}$$

and

$$j_2^z = \psi_2^{\dagger}\gamma^0\gamma^3\psi_2, \tag{37b}$$

in the left and right regions, respectively. Substituting Eqs. (30), (1f) and (3) into these relations yields

$$j_1^z = j_{\mathrm{i}}^z - j_{\mathrm{r}}^z, \tag{38a}$$

with

$$j_{\mathrm{i}}^z = 2\frac{E-qV_1-m}{p_{\mathrm{i}}} \quad \text{and} \quad j_{\mathrm{r}}^z = 2|r|^2\frac{E-qV_1-m}{p_{\mathrm{i}}}, \tag{38b}$$

and

$$j_2^z = j_{\mathrm{t}}^z, \tag{38c}$$

with

$$j_{\mathrm{t}}^z = 2|t|^2\frac{E-qV_2-m}{p_{\mathrm{t}}}. \tag{38d}$$

The reflection and the transmission probabilities are then obtained as

$$R = \left|\frac{j_{\mathrm{r}}^z}{j_{\mathrm{i}}^z}\right| = |r|^2 = \left|\frac{1-\Gamma_{\mathrm{s}}}{1+\Gamma_{\mathrm{s}}}\right|^2 \tag{39a}$$

and

$$T = \left|\frac{j_{\mathrm{t}}^z}{j_{\mathrm{i}}^z}\right| = |t|^2\Gamma_{\mathrm{s}} = \left|\frac{2}{1+\Gamma_{\mathrm{s}}}\right|^2\Gamma_{\mathrm{s}}, \tag{39b}$$

where Γ_{s} was defined in Eqs. (34b). Note that these probabilities verify the probability conservation formula

$$R+T=1. \tag{40}$$

An alternative, pragmatic way to determine the probabilities (39) is to write

$$R = |r|^2\mathscr{C}_R \quad \text{and} \quad T = |t|^2\mathscr{C}_T, \tag{41}$$

where the parameters $\mathscr{C}_R$ and $\mathscr{C}_T$ are "momentum-transition" coefficients, associated with change of region (from V_1 to V_2). Therefore, we must have $\mathscr{C}_R = 1$ and $\mathscr{C}_T \neq 1$. The coefficient $\mathscr{C}_T$ may then be determined from the probability conservation, specifically by inserting $R = |r|^2$ and $T = |t|^2\mathscr{C}_T$ with Eq. (34) into Eq. (40), which leads to $\mathscr{C}_T = \Gamma_{\mathrm{s}}$ and hence retrieves the results in Eqs. (39).

One may distinguish three potential regions in plotting R and T, assuming $(E-qV_1) > m$ so that p_{i} [Eq. (31b)] is real, depending on p_{t} [Eq. (31d)][2]:

1. $qV_2 < E-m$: $p_{\mathrm{t}} =$ real and hence $\Gamma_{\mathrm{s}} = \frac{(E-qV_2-m)\sqrt{(E-qV_1)^2-m^2}}{(E-qV_1-m)\sqrt{(E-qV_2)^2-m^2}}$;

2. $E-m < qV_2 < E+m$: $p_{\mathrm{t}} =$ is imaginary and hence $\Gamma_{\mathrm{s}} = \frac{(E-qV_2-m)\sqrt{(E-qV_1)^2-m^2}}{(E-qV_1-m)(i)\sqrt{-(E-qV_2)^2+m^2}}$;

3. $E+m < qV_2$: $p_{\mathrm{t}} =$ real. However, if we choose $p_{\mathrm{t}} > 0$, then, according to Eq. (31d), $v_{\mathrm{g}} = dE/dp_{\mathrm{t}} = \frac{p_{\mathrm{t}}}{E-qV_2} < 0$ since $E-qV_2 < 0$, which is contradictory to the assumption of positive-z propagation for the transmitted wave. Therefore, in order to maintain a positive group velocity, we must assign a negative sign to p_{t}, viz., use $\Gamma_{\mathrm{s}} = \frac{(E-qV_2-m)\sqrt{(E-qV_1)^2-m^2}}{(E-qV_1-m)(-)\sqrt{(E-qV_2)^2-m^2}}$.

3.2.3 Vector Potential Spatial Step, $A(z)$

The vector potential spatial step, shown in Fig. 1(c), may be written

$$A_3 = A(z) = \begin{cases} A_1 & \text{for } z < z_0, \\ A_2 & \text{for } z > z_0, \end{cases} \quad \text{and} \quad A_0 = 0. \tag{42}$$

Substituting this potential into the solution form (22) and using ansätze corresponding to the related spatial-step electromagnetic solutions[3,6], we assume the left ($z < z_0$) spinor wavefunction

$$\psi_1 = \begin{pmatrix} 1 \\ 0 \\ \frac{E_{\mathrm{i}}-m}{p_{\mathrm{i}}-qA_1} \\ 0 \end{pmatrix} \mathrm{e}^{-iE_{\mathrm{i}}t}\mathrm{e}^{ip_{\mathrm{i}}z} + r \begin{pmatrix} 1 \\ 0 \\ \frac{E_{\mathrm{r}}-m}{p_{\mathrm{r}}-qA_1} \\ 0 \end{pmatrix} \mathrm{e}^{-iE_{\mathrm{r}}t}\mathrm{e}^{ip_{\mathrm{r}}z}, \tag{43a}$$

whose first and second terms correspond to incident and reflected waves, respectively, with reflection amplitude coefficient r, and the transmitted, or right ($z > z_0$), spinor wavefunction

$$\psi_2 = t \begin{pmatrix} 1 \\ 0 \\ \frac{E_\mathrm{t}-m}{p_\mathrm{t}-qA_2} \\ 0 \end{pmatrix} \mathrm{e}^{-iE_\mathrm{t}t}\mathrm{e}^{ip_\mathrm{t}z}, \tag{43b}$$

with transmission amplitude coefficient t.

According to Noether's theorem[7], energy is conserved ($\Delta E = 0$) due to temporal translational symmetry, viz.,

$$E_\mathrm{i} = E_\mathrm{r} = E_\mathrm{t} = E, \tag{44a}$$

whereas the breaking of spatial symmetry implies momentum transformations, which are found from Eq. (18) with $A_3 = A(z)$ and $A_0 = V = 0$ to be

$$p_\mathrm{i} = \sqrt{E^2 - m^2} + qA_1, \tag{44b}$$

$$p_\mathrm{r} = -p_\mathrm{i} + 2qA_1 \tag{44c}$$

and

$$p_\mathrm{t} = \sqrt{E^2 - m^2} + qA_2, \tag{44d}$$

where the apparent negative momentum in Eq. (44c) simply represents propagation in the negative z direction, with positive momentum.

According to Eq. (8), the spinor wavefunction must be continuous at the spatial discontinuity, viz.,

$$\psi_1|_{z=z_0} = \psi_2|_{z=z_0}. \tag{45}$$

Inserting Eqs. (43) in this relation yields

$$1 + r = t \tag{46a}$$

and

$$\frac{E_\mathrm{i} - m}{p_\mathrm{i} - qA_1} + r\frac{E_\mathrm{r} - m}{p_\mathrm{r} - qA_1} = t\frac{E_\mathrm{t} - m}{p_\mathrm{t} - qA_2}. \tag{46b}$$

Substituting then Eqs. (44) into Eq. (46b), and solving the system formed by the resulting equation and Eq. (46a), finally leads, after some algebraic manipulations, to

$$r = 0 \quad \text{and} \quad t = 1, \tag{47}$$

which reveals that the vector potential spatial step, $A(z)$, does not produce any back-scattering.

3.2.4 Vector Potential Temporal Step, $A(t)$

The vector potential temporal step, shown in Fig. 1(d), may be written

$$A_3 = A(t) = \begin{cases} A_1 & \text{for } t < t_0, \\ A_2 & \text{for } t > t_0, \end{cases} \quad \text{and} \quad A_0 = V = 0. \tag{48}$$

Substituting this potential into the solution form (22) and using ansätze corresponding to the related temporal-step electromagnetic solutions[5,6], we assume the incident, or earlier ($t < t_0$), spinor wavefunction

$$\psi_1 = \begin{pmatrix} 1 \\ 0 \\ \frac{E_\mathrm{i}-m}{p_\mathrm{i}-qA_1} \\ 0 \end{pmatrix} \mathrm{e}^{-iE_\mathrm{i}t}\mathrm{e}^{ip_\mathrm{i}z} \tag{49a}$$

and the later ($t > t_0$) spinor wavefunction

$$\psi_2 = f \begin{pmatrix} 1 \\ 0 \\ \frac{E_\mathrm{f}-m}{p_\mathrm{f}-qA_2} \\ 0 \end{pmatrix} \mathrm{e}^{-iE_\mathrm{f}t}\mathrm{e}^{ip_\mathrm{f}z} + b \begin{pmatrix} 1 \\ 0 \\ \frac{E_\mathrm{b}-m}{p_\mathrm{b}-qA_2} \\ 0 \end{pmatrix} \mathrm{e}^{-iE_\mathrm{b}t}\mathrm{e}^{ip_\mathrm{b}z}, \tag{49b}$$

whose first and second terms correspond to later-forward and later-backward waves, respectively, with corresponding amplitude coefficients f and b.

According to Noether's theorem[7], momentum is conserved ($\Delta p = 0$) due to spatial translational symmetry, viz.,

$$p_\mathrm{i} = p_\mathrm{f} = p_\mathrm{b} = p, \tag{50a}$$

whereas the breaking of spatial symmetry implies energy transformations, which are found from Eq. (18) with $A_3 = A(t)$ and $A_0 = V = 0$ to be

$$E_\mathrm{i} = \sqrt{(p - qA_1)^2 + m^2}, \tag{50b}$$

$$E_\mathrm{f} = \sqrt{(p - qA_2)^2 + m^2} \tag{50c}$$

and

$$E_\mathrm{b} = -E_\mathrm{f}, \tag{50d}$$

where the apparent negative energy in the last relation simply represents propagation in the negative z direction, with positive energy.

According to Eq. (8), the spinor wavefunction must be continuous at the temporal discontinuity, viz.,

$$\psi_1|_{t=t_0} = \psi_2|_{t=t_0}. \tag{51}$$

Inserting Eqs. (24) into this relation yields

$$1 = f + b \tag{52a}$$

and

$$\frac{E_\mathrm{i} - m}{p_\mathrm{i} - qA_1} = f\frac{E_\mathrm{f} - m}{p_\mathrm{f} - qA_2} + b\frac{E_\mathrm{b} - m}{p_\mathrm{b} - qA_2}. \tag{52b}$$

Substituting then Eqs. (50) into Eq. (52b), and solving the system formed by the resulting equation and Eq. (52a), finally leads, after some algebraic manipulations, to

$$f = \frac{1 + \Gamma_\mathrm{t}}{2\Gamma_\mathrm{t}} \quad \text{and} \quad b = \frac{\Gamma_\mathrm{t} - 1}{2\Gamma_\mathrm{t}} \tag{53a}$$

where

$$\Gamma_\mathrm{t} = \frac{\dfrac{E_\mathrm{f}}{p - qA_2}}{\dfrac{E_\mathrm{i} - m}{p - qA_1} + \dfrac{m}{p - qA_2}}. \tag{53b}$$

This expression may be alternatively written in terms of E_i and $A_{1,2}$ only upon first using Eq. (50c) to eliminate E_f and then substituting in the resulting expression

$$p = \sqrt{E_\mathrm{i}^2 - m^2} + qA_1, \tag{53c}$$

which was obtained from Eq. (50c), which yields

$$\Gamma_\mathrm{t} = \frac{\sqrt{\left(\sqrt{E_\mathrm{i}^2 - m^2} - (qA_2 - qA_1)\right)^2 + m^2}}{\left(\sqrt{E_\mathrm{i}^2 - m^2} - (qA_2 - qA_1)\right)\left(\dfrac{E_\mathrm{i} - m}{\sqrt{E_\mathrm{i}^2 - m^2}}\right) + m}. \tag{53d}$$

The probabilities associated with the later-forward and later-backward waves cannot be found from the ratios of the corresponding Dirac currents, contrary to the case of the $V(z)$ problem in Sec. 3.2.1, because the Dirac current is *not* conserved here, due to the non-conservation of energy. However, we may resort to an alternative approach similar to that also used in Sec. 3.2.1, writing

$$F = |f|^2 \mathscr{C}_F \quad \text{and} \quad B = |b|^2 \mathscr{C}_B, \tag{54}$$

where the parameters $\mathscr{C}_F$ and $\mathscr{C}_B$ are now "energy-transition" coefficients, associated with change of region (from A_1 to A_2). Therefore, we must have $\mathscr{C}_F = \mathscr{C}_B = \mathscr{C}$, since the two probabilities correspond to the same change of region, from the earlier region (A_1) to the later region (A_2), so that

$$F = |f|^2 \mathscr{C} \quad \text{and} \quad B = |b|^2 \mathscr{C}. \tag{55}$$

At the same, probability must be conserved, since the particle can only either keep moving forward or move backward, viz.,

$$F + B = 1. \tag{56}$$

Substituting then Eqs. (55) with Eqs. (53a) into Eq. (56) yields then

$$\mathscr{C} = \frac{2\Gamma_\mathrm{t}^2}{1 + \Gamma_\mathrm{t}^2}, \tag{57}$$

so that the later forward and backward probabilities are finally obtained from Eq. (55) as

$$F = |f|^2 \frac{2\Gamma_\mathrm{t}^2}{1 + \Gamma_\mathrm{t}^2} = \left|\frac{1 + \Gamma_\mathrm{t}}{2\Gamma_\mathrm{t}}\right|^2 \frac{2\Gamma_\mathrm{t}^2}{1 + \Gamma_\mathrm{t}^2} \tag{58a}$$

and

$$B = |b|^2 \frac{2\Gamma_\mathrm{t}^2}{1 + \Gamma_\mathrm{t}^2} = \left|\frac{\Gamma_\mathrm{t} - 1}{2\Gamma_\mathrm{t}}\right|^2 \frac{2\Gamma_\mathrm{t}^2}{1 + \Gamma_\mathrm{t}^2}, \tag{58b}$$

where Eq. (53a) was used in the second equalities and where Γ_t was defined in Eq. (53d).

4 Gauge Transformations and Symmetries

4.1 Scalar Potential Spatial Step $V(z)$

The electric and magnetic fields, **E** and **B**, are generally related to the potentials as

$$\mathbf{E} = -\nabla V - \frac{\partial \mathbf{A}}{\partial t} \tag{59a}$$

and

$$\mathbf{B} = \nabla \times \mathbf{A}, \tag{59b}$$

which are invariant under the gauge transformation[3,8]

$$V \to V' = V - \frac{\partial \Lambda}{\partial t} \tag{60a}$$

and

$$\mathbf{A} \to \mathbf{A}' = \mathbf{A} + \nabla \Lambda, \tag{60b}$$

where Λ is an arbitrary scalar function. The potential spatial step $V(z)$ is equivalent to the transformation

$$V' = V_1 + \Delta V \theta(z - z_0) \quad \text{and} \quad \mathbf{A}' = 0, \tag{61}$$

Consistency with the gauge (60) would demand that

$$\frac{\partial \Lambda}{\partial t} = -\Delta V \theta(z - z_0) \quad \text{and} \quad \nabla \Lambda = 0. \tag{62}$$

which imply

$$\Lambda = -\Delta V t \theta(z - z_0) \quad \text{and} \quad \Lambda \neq \Lambda(z). \tag{63}$$

respectively. The incompatibility between these two conditions on Λ indicates that the transformation indeed breaks the symmetry of the gauge (60), which explains why the corresponding potential leads to electron back-scattering[9] (Sec. 4.1).

4.2 Vector Potential Spatial Step $A(z)$

The vector potential spatial step $\mathbf{A}(z) = A(z)\hat{\mathbf{z}}$ is equivalent to the transformation

$$V' = 0 \quad \text{and} \quad \mathbf{A}' = A_1 + \Delta A \theta(z - z_0), \tag{64}$$

which is a particular case of the gauge transformation (60) with

$$\frac{\partial \Lambda}{\partial t} = 0 \quad \text{and} \quad \nabla \Lambda = \Delta A \theta(z - z_0), \tag{65}$$

corresponding to

$$\Lambda = \Delta A \theta(z - z_0) z = \Lambda(z) \tag{66}$$

Therefore, the step $A(z)$ does not involve any change in the fields, and hence also in the impedance, which explains why we found that it produces no (reflected wave) back-scattering (Sec. 3.2.3).

5 Phase and Group Velocities

The phase and group velocities may be computed from the dispersion relation (18), i.e.,

$$(E-qA_0)^2=(p-qA_3)^2+m^2. \tag{67}$$

The phase velocity is defined as

$$v_\mathrm{p}=\frac{E}{p}. \tag{68}$$

In general, it may be found by solving Eq. (67) for p and substituting the result into Eq. (68), which yields

$$v_\mathrm{p}=\frac{E}{\sqrt{(E-qA_0)^2-m^2}+qA_3}. \tag{69}$$

For the cases of the scalar potential spatial step $[V(z)]$ (Sec. 3.2.2) and the vector potential temporal step $[A(t)]$ (Sec. 3.2.4), the scattered phase velocities may be directly obtained from Eq. (68) as

$$v_\mathrm{p,t}=\frac{E}{p_\mathrm{t}} \quad \text{and} \quad v_\mathrm{p.r}=\frac{E}{p_\mathrm{r}}, \tag{70}$$

with p_t and p_r given by Eq. (31d) and Eq. (31b), and

$$v_\mathrm{p,f}=\frac{E_\mathrm{f}}{p} \quad \text{and} \quad v_\mathrm{p,b}=\frac{E_\mathrm{b}}{p}, \tag{71}$$

with E_f and E_b given by Eq. (50c) and Eq. (50d), respectively.

The group velocity is defined as

$$v_\mathrm{g}=\frac{\partial E}{\partial p}. \tag{72}$$

Its general expression may be found by taking the derivative of Eq. (67) versus p and isolating $\partial E/\partial p$, which results in

$$v_\mathrm{g}=\frac{p-qA_3}{E-qA_0}. \tag{73}$$

For the cases of the scalar potential spatial step $[V(z)]$ (Sec. 3.2.2) and the vector potential temporal step $[A(t)]$ (Sec. 3.2.4), the scattered group velocities may be obtained from Eq. (73) as

$$v_\mathrm{g,t}=\frac{p_\mathrm{t}}{E-qV} \quad \text{and} \quad v_\mathrm{g,r}=\frac{p_\mathrm{r}}{E-qV}, \tag{74}$$

with p_t and p_r given by Eq. (31d) and Eq. (31b), and

$$v_\mathrm{g,f}=\frac{p-qA}{E_\mathrm{f}} \quad \text{and} \quad v_\mathrm{g,b}=\frac{p-qA}{E_\mathrm{b}}, \tag{75}$$

with E_f and E_b given by Eq. (50c) and Eq. (50d), respectively.

In the problem of the scalar potential spatial step $[V(z)]$ (Sec. 3.2.2), assuming $qV_1<E_\mathrm{i}-m$ and $qV_1<qV_2$, we find, using Eqs. (70) and Eq. (74)

1. $qV_2<E-m\rightarrow v_{\mathrm{g},2}<v_{\mathrm{g},1}$ and $v_{\mathrm{p},1}<v_{\mathrm{p},2}$,
2. $E-m<qV_2<E+m\rightarrow v_{\mathrm{g},2}=0$ and $v_{\mathrm{p},2}=\infty$ (Klein region),
3. $E+m<qV_2<2E-V_1\rightarrow v_{\mathrm{g},2}<v_{\mathrm{g},1}$ and $v_{\mathrm{p},1}<v_{\mathrm{p},2}$,
4. $2E-V_1<V_2\rightarrow v_{\mathrm{g},1}<v_{\mathrm{g},2}$ and $v_{\mathrm{p},2}<v_{\mathrm{p},1}$,

while in the problem of the vector potential temporal step $[A(t)]$ (Sec. 3.2.4), assuming $qA_1<p_\mathrm{i}$ and $qA_1<qA_2$, we find, using Eqs. (71) and Eq. (75),

1. $qA_2<p_\mathrm{i}\rightarrow v_{\mathrm{g},2}<v_{\mathrm{g},1}$ and $v_{\mathrm{p},2}<v_{\mathrm{p},1}$,
2. $p_\mathrm{i}<qA_2<2p_\mathrm{i}-A_1\rightarrow v_{\mathrm{g},2}<v_{\mathrm{g},1}$ and $v_{\mathrm{p},2}<v_{\mathrm{p},1}$,
3. $2p_\mathrm{i}-A_1<qA_2\rightarrow v_{\mathrm{g},1}<v_{\mathrm{g},2}$ and $v_{\mathrm{p},1}<v_{\mathrm{p},2}$,

where 1 and 2 refer to the two regions.

6 Spatial and Temporal Step Electromagnetic Problems

We provide here the main results pertaining to the 1+1D spatial and temporal step electromagnetic problems for the sake of comparison. Related details are available in[6,10].

6.1 Spatial Step Problem

The wave equation may be written as

$$\left[\frac{1}{n^2}\frac{\partial^2}{\partial z^2}-\frac{\partial^2}{\partial t^2}\right]\left\{\begin{array}{c}E(z,t)\\H(z,t)\end{array}\right\}=0. \tag{76}$$

The E and H fields before ($z<z_0$, region 1) and after ($z>z_0$, region 2) the step are

$$E_1=\mathrm{e}^{-i\omega_\mathrm{i}t}\mathrm{e}^{ik_\mathrm{i}z}+r\mathrm{e}^{-i\omega_\mathrm{r}t}\mathrm{e}^{ik_\mathrm{r}z}, \tag{77a}$$

$$H_1=\left(\mathrm{e}^{-i\omega_\mathrm{i}t}\mathrm{e}^{ik_\mathrm{i}z}-r\mathrm{e}^{-i\omega_\mathrm{r}t}\mathrm{e}^{ik_\mathrm{r}z}\right)/\eta_1, \tag{77b}$$

$$E_2=t\mathrm{e}^{-i\omega_\mathrm{t}t}\mathrm{e}^{ik_\mathrm{t}z}, \tag{77c}$$

$$H_2=t\mathrm{e}^{-i\omega_\mathrm{t}t}\mathrm{e}^{ik_\mathrm{t}z}/\eta_2, \tag{77d}$$

where

$$\eta_{1,2}=\sqrt{\frac{\mu_{1,2}}{\varepsilon_{1,2}}} \tag{78}$$

is the intrinsic medium impedance, and

$$\omega_\mathrm{i}=\omega_\mathrm{r}=\omega_\mathrm{t}, \tag{79a}$$

$$k_\mathrm{i}=-k_\mathrm{r}=n_1\frac{\omega_\mathrm{i}}{c},\quad\text{and}\quad k_\mathrm{t}=n_2\frac{\omega_\mathrm{i}}{c}. \tag{79b}$$

Inserting Eqs. (77) to (79) into the boundary condition relation

$$E_1|_{z=z_0}=E_2|_{z=z_0}\quad\text{and}\quad H_1|_{z=z_0}=H_2|_{z=z_0} \tag{80}$$

yields then the scattering amplitude coefficients

$$r=\frac{1-N}{1+N}\quad\text{and}\quad t=\frac{2}{1+N}, \tag{81}$$

while the reflectance and transmittance are found, using the Poynting vector definitions

$$R=\left|\frac{\mathbf{E}_\mathrm{r}\times\mathbf{H}_\mathrm{r}}{\mathbf{E}_\mathrm{i}\times\mathbf{H}_\mathrm{i}}\right|\quad\text{and}\quad T=\left|\frac{\mathbf{E}_\mathrm{t}\times\mathbf{H}_\mathrm{t}}{\mathbf{E}_\mathrm{i}\times\mathbf{H}_\mathrm{i}}\right|, \tag{82}$$

and substituting Eqs. (77) in these relations, as

$$R=|r|^2=\left|\frac{1-N}{1+N}\right|^2\quad\text{and}\quad T=|t|^2N=\left|\frac{2}{1+N}\right|^2N, \tag{83}$$

where $N=\dfrac{n_2}{n_1}=\dfrac{\eta_1}{\eta_2}$, assuming nonmagnetic materials.

The space-time and dispersion diagrams corresponding to these results are provided in Fig. 2(a).

6.2 Temporal Step Problem

The wave equation may be written as

$$\left[\frac{\partial^2}{\partial z^2} - n^2 \frac{\partial^2}{\partial t^2}\right] \left\{ \begin{array}{c} D(z,t) \\ B(z,t) \end{array} \right\} = 0. \tag{84}$$

The D and B fields before ($t < t_0$, region 1) and after ($t > t_0$, region 2) the step are

$$D_1 = \mathrm{e}^{-i\omega_\mathrm{i} t} \mathrm{e}^{ik_\mathrm{i} z}, \tag{85a}$$

$$B_1 = \left(\mathrm{e}^{-i\omega_\mathrm{i} t} \mathrm{e}^{ik_\mathrm{i} z}\right) / \eta_1, \tag{85b}$$

$$D_2 = f \mathrm{e}^{-i\omega_\mathrm{f} t} \mathrm{e}^{ik_\mathrm{f} z} + b \mathrm{e}^{-i\omega_\mathrm{b} t} \mathrm{e}^{ik_\mathrm{b} z}, \tag{85c}$$

$$B_2 = \left(f \mathrm{e}^{-i\omega_\mathrm{f} t} \mathrm{e}^{ik_\mathrm{f} z} - b \mathrm{e}^{-i\omega_\mathrm{b} t} \mathrm{e}^{ik_\mathrm{b} z}\right) / \eta_2, \tag{85d}$$

and

$$k_\mathrm{i} = k_\mathrm{f} = k_\mathrm{b}, \tag{86a}$$

$$\omega_\mathrm{i} = \frac{c}{n_1} k_\mathrm{i} \quad \text{and} \quad \omega_\mathrm{f} = -\omega_\mathrm{b} = \frac{c}{n_2} k_\mathrm{i}. \tag{86b}$$

Inserting Eqs. (85) to (86) into the boundary condition relation

$$D_1|_{t=t_0} = D_2|_{t=t_0} \quad \text{and} \quad B_1|_{t=t_0} = B_2|_{t=t_0}, \tag{87}$$

yields then the scattering amplitude coefficients

$$f = \frac{1+N}{2N} \quad \text{and} \quad b = \frac{N-1}{2N}, \tag{88}$$

while the reflectance and transmittance are found, using the Poynting vector definitions

$$F = \left|\frac{\mathbf{E}_\mathrm{f} \times \mathbf{H}_\mathrm{f}}{\mathbf{E}_\mathrm{i} \times \mathbf{H}_\mathrm{i}}\right| = \left|\frac{\mathbf{D}_\mathrm{f}/\varepsilon_2 \times \mathbf{B}_\mathrm{f}/\mu_2}{\mathbf{D}_\mathrm{i}/\varepsilon_1 \times \mathbf{B}_\mathrm{i}/\mu_1}\right| = \left|\frac{\mathbf{D}_\mathrm{f} \times \mathbf{B}_\mathrm{f}}{\mathbf{D}_\mathrm{i} \times \mathbf{B}_\mathrm{i}}\right| \left(\frac{n_1}{n_2}\right)^2 \tag{89a}$$

$$B = \left|\frac{\mathbf{E}_\mathrm{b} \times \mathbf{H}_\mathrm{b}}{\mathbf{E}_\mathrm{i} \times \mathbf{H}_\mathrm{i}}\right| = \left|\frac{\mathbf{D}_\mathrm{b}/\varepsilon_2 \times \mathbf{B}_\mathrm{b}/\mu_2}{\mathbf{D}_\mathrm{i}/\varepsilon_1 \times \mathbf{B}_\mathrm{i}/\mu_1}\right| = \left|\frac{\mathbf{D}_\mathrm{b} \times \mathbf{B}_\mathrm{b}}{\mathbf{D}_\mathrm{i} \times \mathbf{B}_\mathrm{i}}\right| \left(\frac{n_1}{n_2}\right)^2, \tag{89b}$$

and substituting Eqs. (85) in these relations, as

$$F = |f|^2 \frac{1}{N} = \left|\frac{1+N}{2N}\right|^2 \frac{1}{N} \quad \text{and} \quad B = |b|^2 \frac{1}{N} = \left|\frac{N-1}{2N}\right|^2 \frac{1}{N}, \tag{90}$$

where $N = \frac{n_2}{n_1} = \frac{\eta_1}{\eta_2}$, assuming nonmagnetic materials.

The space-time and dispersion diagrams corresponding to these results are provided in Fig. 2(b).

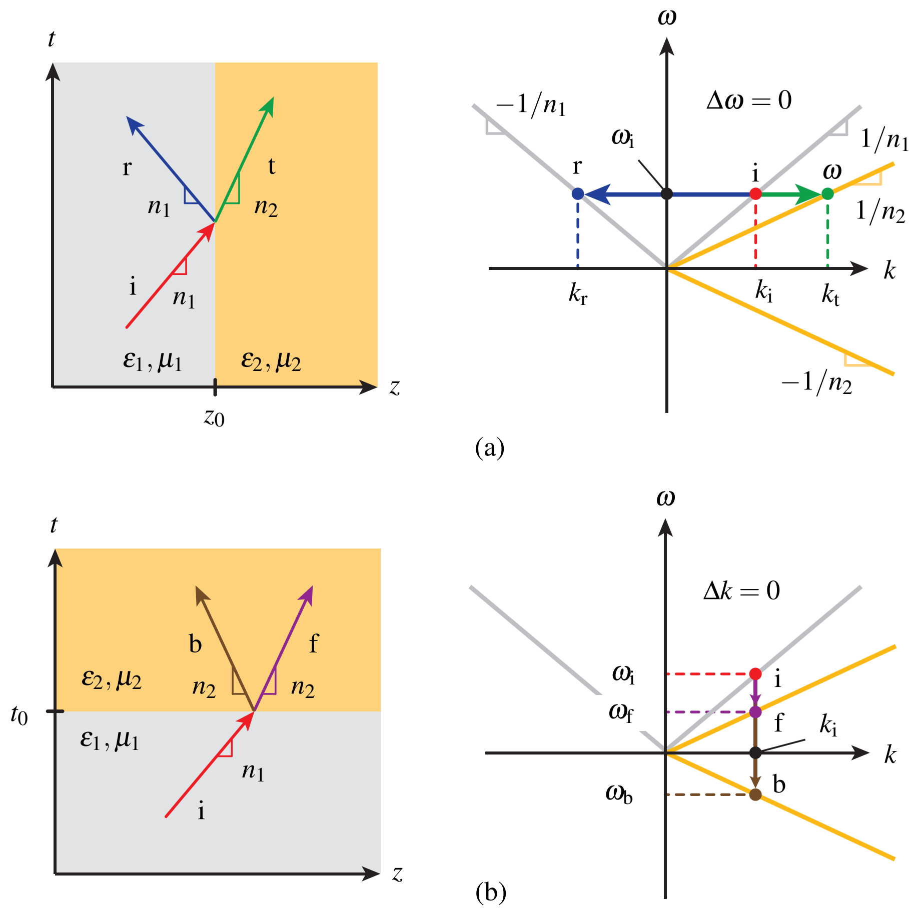

Figure 2. Electromagnetic wave scattering at (a) a spatial and (b) a temporal refractive index step discontinuity, represented in space-time diagrams (left panels) and dispersion diagrams with transitions (right panels).

7 Smooth Temporal Step

7.1 Dirac Equation for a Time-Varying Vector Potential

We start with the Dirac equation in its covariant form[2], viz.,

$$\left[\gamma^\mu (i\partial_\mu - qA_\mu) - m\right] \psi = 0, \tag{91a}$$

which involves the four-gradient

$$\partial_\mu \equiv (\partial_0, \partial_i), \quad \text{with} \quad i = 1,2,3, \tag{91b}$$

and the gamma matrices[1]

$$\gamma^0 = \begin{pmatrix} 0 & I \\ I & 0 \end{pmatrix}, \quad \text{with} \quad I = \begin{pmatrix} 1 & 0 \\ 0 & 1 \end{pmatrix}, \tag{91c}$$

and

$$\gamma^i = \begin{pmatrix} 0 & -\sigma^i \\ \sigma^i & 0 \end{pmatrix}, \tag{91d}$$

with the Pauli matrices

$$\sigma^1 = \begin{pmatrix} 0 & 1 \\ 1 & 0 \end{pmatrix}, \quad \sigma^2 = \begin{pmatrix} 0 & -i \\ i & 0 \end{pmatrix} \quad \text{and} \quad \sigma^3 = \begin{pmatrix} 1 & 0 \\ 0 & -1 \end{pmatrix}. \tag{91e}$$

In the sequel of this section, we assume a 1+1D (t and z) spacetime system and a z-directed magnetic vector potential, viz., $A_0 = A_1 = A_2 = 0$ and $A_3 = A_z(t)$. These assumption reduce Eq. (91a) to

$$\left[\gamma^0 i\partial_0 + \gamma^3 (i\partial_3 + qA_z(t)) - m\right] \psi = 0, \tag{92}$$

which, using the spinor ansatz

$$\psi = \begin{pmatrix} \varphi \\ \vartheta \end{pmatrix} e^{ipz}, \tag{93}$$

takes the more explicit form

$$\left[\gamma^0 i\partial_0 - \gamma^3 (p - qA_z(t)) - m\right] \begin{pmatrix} \varphi \\ \vartheta \end{pmatrix} = 0. \tag{94}$$

Inserting the gamma matrices (91c) and (91d) with the sigma matrices (91e) into Eq. (94) yields then

$$\left[\begin{pmatrix} 0 & 1 \\ 1 & 0 \end{pmatrix} i\partial_0 - \begin{pmatrix} 0 & -1 \\ 1 & 0 \end{pmatrix} (p - qA_z(t)) - m\right] \begin{pmatrix} \varphi \\ \vartheta \end{pmatrix} = 0, \tag{95}$$

which splits into the two equations

$$i\frac{\mathrm{d}\vartheta}{\mathrm{d}t} + (p - qA_z(t))\vartheta - m\varphi = 0 \tag{96a}$$

and

$$i\frac{\mathrm{d}\varphi}{\mathrm{d}t} - (p - qA_z(t))\varphi - m\vartheta = 0. \tag{96b}$$

Isolating ϑ in Eq. (96b),

$$\vartheta = \frac{1}{m}\left[i\frac{\mathrm{d}\varphi}{\mathrm{d}t} - (p - qA_z(t))\varphi\right], \tag{97}$$

[1] Note that we have chosen here the Weyl representation for these matrices[2]. Other choices would have been the Dirac and Majorana representations[2]. The reason for that choice is that it leads – as we found in painstaking derivations – to convenient hypergeometric expressions and ultimately to closed-form solutions to the problem.

and substituting this expression as well as its time derivative,

$$\frac{\mathrm{d}\vartheta}{\mathrm{d}t} = \frac{1}{m}\left[i\frac{\mathrm{d}^2\varphi}{\mathrm{d}t^2} + q\frac{\mathrm{d}A_z(t)}{\mathrm{d}t}\varphi - (p - qA_z(t))\frac{\mathrm{d}\varphi}{\mathrm{d}t}\right], \tag{98}$$

into Eq. (96a), we get

$$\frac{\mathrm{d}^2\varphi}{\mathrm{d}t^2} + \left[(p - qA_z(t))^2 + m^2 - iq\frac{\mathrm{d}A_z(t)}{\mathrm{d}t}\right]\varphi = 0. \tag{99}$$

Now, we select for the potential $A_z(t)$ the smooth vector potential corresponding to the hyperbolic tangent function

$$A_z(t) = A_1 + \frac{A_2 - A_1}{2}\left[1 + \tanh\left(\frac{t - t_0}{\tau}\right)\right] = \frac{A_1 + A_2 \mathrm{e}^{2\frac{t-t_0}{\tau}}}{1 + \mathrm{e}^{2\frac{t-t_0}{\tau}}}, \tag{100}$$

which corresponds to the electric field

$$E_z(t) = -\frac{\partial A_z(t)}{\partial t} = -\frac{(A_2 - A_1)}{2\tau}\operatorname{sech}^2\left(\frac{t - t_0}{\tau}\right) = -\frac{2(A_2 - A_1)}{\tau}\frac{\mathrm{e}^{2\frac{t-t_0}{\tau}}}{\left(1 + \mathrm{e}^{2\frac{t-t_0}{\tau}}\right)^2}, \tag{101}$$

where τ is a time constant.

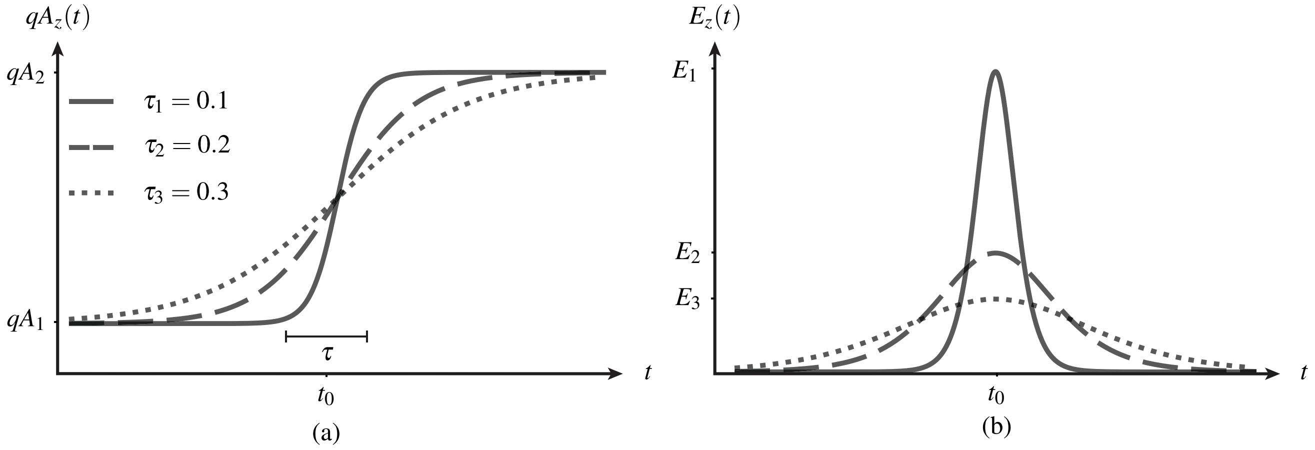

Figure 3. Functions associated with the smooth potential investigated in Sec. 7 for different values of the time parameter τ. (a) Potential function itself, $A_z(t)$ [Eq. (100)]. (b) Corresponding electric field, $E_z(t)$ [Eq. (101)].

We shall next solve Eq. (99) for the earlier medium and later medium separately so as to be able to subsequently apply boundary conditions between them. For each case, we shall define a new variable that will simplify the vector potential in Eq. (100) to the point that Eq. (99) will progressively transform into a hypergeometric equation, which admits analytical solutions.

7.2 Solution for the Earlier Medium ($t < t_0$)

For the earlier (e) medium, we use the variable

$$\zeta_\mathrm{e} = -\mathrm{e}^{2\frac{t-t_0}{\tau}}, \tag{102}$$

which simplifies Eq. (100) to

$$A_z(\zeta_\mathrm{e}) = \frac{A_1 - A_2\zeta_\mathrm{e}}{1 - \zeta_\mathrm{e}}, \tag{103a}$$

and whose first and second time derivatives lead to

$$\frac{\mathrm{d}}{\mathrm{d}t} = \frac{2}{\tau}\zeta_{\mathrm{e}}\frac{\mathrm{d}}{\mathrm{d}\zeta_{\mathrm{e}}}, \tag{103b}$$

and

$$\frac{\mathrm{d}^2}{\mathrm{d}t^2} = \frac{4}{\tau^2}\zeta_{\mathrm{e}}\frac{\mathrm{d}}{\mathrm{d}\zeta_{\mathrm{e}}} + \frac{4}{\tau^2}\zeta_{\mathrm{e}}^2\frac{\mathrm{d}^2}{\mathrm{d}\zeta_{\mathrm{e}}^2}, \tag{103c}$$

respectively.

Inserting Eqs. (103) into Eq. (99) and multiplying the result by $\frac{\tau^2}{4}\frac{1-\zeta_{\mathrm{e}}}{\zeta_{\mathrm{e}}}$, we get, after some algebraic manipulations,

$$\zeta_{\mathrm{e}}(1-\zeta_{\mathrm{e}})\frac{\mathrm{d}^2\varphi_{\mathrm{e}}}{\mathrm{d}\zeta_{\mathrm{e}}^2} + (1-\zeta_{\mathrm{e}})\frac{\mathrm{d}\varphi_{\mathrm{e}}}{\mathrm{d}\zeta_{\mathrm{e}}} + \frac{1}{\zeta_{\mathrm{e}}(1-\zeta_{\mathrm{e}})}\left[\alpha_2\zeta_{\mathrm{e}}^2 + \alpha_0\zeta_{\mathrm{e}} + \alpha_1\right]\varphi_{\mathrm{e}} = 0, \tag{104a}$$

where

$$\alpha_0 = -\frac{\tau^2}{4}\left[((p-qA_2)^2+m^2)+((p-qA_1)^2+m^2)-(qA_2-qA_1)^2-2i\frac{qA_2-qA_1}{\tau}\right], \tag{104b}$$

$$\alpha_1 = \frac{\tau^2}{4}\left[(p-qA_1)^2+m^2\right] = \frac{\tau^2}{4}E_1^2, \tag{104c}$$

and

$$\alpha_2 = \frac{\tau^2}{4}\left[(p-qA_2)^2+m^2\right] = \frac{\tau^2}{4}E_2^2, \tag{104d}$$

and whose singularities at $\zeta_{\mathrm{e}} = 0$ and $1-\zeta_{\mathrm{e}} = 0$ can be removed by inserting the expression

$$\varphi_{\mathrm{e}}(\zeta_{\mathrm{e}}) = \zeta_{\mathrm{e}}^{\mu}(1-\zeta_{\mathrm{e}})^{\nu}f(\zeta_{\mathrm{e}}), \tag{105}$$

its derivative,

$$\frac{\mathrm{d}\varphi_{\mathrm{e}}}{\mathrm{d}\zeta_{\mathrm{e}}} = \zeta_{\mathrm{e}}^{\mu}(1-\zeta_{\mathrm{e}})^{\nu}\left[\left(\mu\frac{1}{\zeta_{\mathrm{e}}} - \nu\frac{1}{1-\zeta_{\mathrm{e}}}\right)f + \frac{\mathrm{d}f}{\mathrm{d}\zeta_{\mathrm{e}}}\right], \tag{106a}$$

and its second derivative,

$$\frac{\mathrm{d}^2\varphi_{\mathrm{e}}}{\mathrm{d}\zeta_{\mathrm{e}}^2} = \zeta_{\mathrm{e}}^{\mu}(1-\zeta_{\mathrm{e}})^{\nu}\left[\left(\mu(\mu-1)\frac{1}{\zeta_{\mathrm{e}}^2} + \nu(\nu-1)\frac{1}{(1-\zeta_{\mathrm{e}})^2} - 2\mu\nu\frac{1}{\zeta_{\mathrm{e}}(1-\zeta_{\mathrm{e}})}\right)f + \left(2\mu\frac{1}{\zeta_{\mathrm{e}}} - 2\nu\frac{1}{1-\zeta_{\mathrm{e}}}\right)\frac{\mathrm{d}f}{\mathrm{d}\zeta_{\mathrm{e}}} + \frac{\mathrm{d}^2 f}{\mathrm{d}\zeta_{\mathrm{e}}^2}\right]. \tag{106b}$$

Dividing the resulting equation by $\zeta_{\mathrm{e}}^{\mu}(1-\zeta_{\mathrm{e}})^{\nu}$ finally yields the hypergeometric-type equation

$$\zeta_{\mathrm{e}}(1-\zeta_{\mathrm{e}})\frac{\mathrm{d}^2 f}{\mathrm{d}\zeta_{\mathrm{e}}^2} + \left[(2\mu+1)-(2\mu+2\nu+1)\zeta_{\mathrm{e}}\right]\frac{\mathrm{d}f}{\mathrm{d}\zeta_{\mathrm{e}}} - \left[(\mu+\nu+\lambda)(\mu+\nu-\lambda)\right]f = 0, \tag{107a}$$

where

$$\mu = i\sqrt{\alpha_1} = i\frac{\tau}{2}E_1, \tag{107b}$$

$$\nu = \frac{1-\sqrt{1-4(\alpha_0+\alpha_1+\alpha_2)}}{2} = i\frac{\tau}{2}(qA_2-qA_1), \tag{107c}$$

and

$$\lambda = i\sqrt{\alpha_2} = i\frac{\tau}{2}E_2. \tag{107d}$$

The solution to Eqs. (107) can be expressed in terms of Gauss hypergeometric functions ${}_2F_1(\cdot)$[11] as

$$f(\zeta_e) = C_1 {}_2F_1(\mu+\nu-\lambda, \mu+\nu+\lambda; 1+2\mu; \zeta_e) + C_2 \zeta_e^{-2\mu} {}_2F_1(-\mu+\nu-\lambda, -\mu+\nu+\lambda; 1-2\mu; \zeta_e), \tag{108}$$

and substituting this equation into Eq. (105) allows to determine the wavefunction $\varphi_e(\zeta_e)$ in terms of Gauss hypergeometric functions as

$$\begin{aligned}\varphi_e(\zeta_e) = {} & C_1 \zeta_e^{\mu}(1-\zeta_e)^{\nu} {}_2F_1(\mu+\nu-\lambda, \mu+\nu+\lambda; 1+2\mu; \zeta_e) \\ & + C_2 \zeta_e^{-\mu}(1-\zeta_e)^{\nu} {}_2F_1(-\mu+\nu-\lambda, -\mu+\nu+\lambda; 1-2\mu; \zeta_e).\end{aligned} \tag{109}$$

Since there is only an incident wave in the earlier medium, considering Eq. (107b), the coefficient C_1 must be zero, which reduces Eq. (109) to

$$\varphi_e(\zeta_e) = C_2 \zeta_e^{-\mu}(1-\zeta_e)^{\nu} {}_2F_1(-\mu+\nu-\lambda, -\mu+\nu+\lambda; 1-2\mu; \zeta_e). \tag{110}$$

To complete the spinor wavefunction, we also need to calculate ϑ_e. This may be accomplished by inserting Eq. (110) into Eq. (97) and using Eqs. (103a) and (103b). This includes the intermediate step

$$\begin{aligned}\frac{d\varphi_e}{d\zeta_e} = {} & -C_2 \mu \zeta_e^{-\mu-1}(1-\zeta_e)^{\nu} {}_2F_1(-\mu+\nu-\lambda, -\mu+\nu+\lambda; 1-2\mu; \zeta_e) \\ & - C_2 \zeta_e^{-\mu} \nu (1-\zeta_e)^{\nu-1} {}_2F_1(-\mu+\nu-\lambda, -\mu+\nu+\lambda; 1-2\mu; \zeta_e) \\ & + C_2 \zeta_e^{-\mu}(1-\zeta_e)^{\nu} \frac{(-\mu+\nu-\lambda)(-\mu+\nu+\lambda)}{1-2\mu} {}_2F_1(-\mu+\nu-\lambda+1, -\mu+\nu+\lambda+1; 1-2\mu+1; \zeta_e),\end{aligned} \tag{111}$$

where using the derivative property of hypergeometric functions[11],

$$\frac{d}{d\zeta}[{}_2F_1(a,b;c;\zeta)] = \frac{ab}{c} {}_2F_1(a+1, b+1; c+1, \zeta), \tag{112}$$

leads to

$$\begin{aligned}\vartheta_e = \frac{C_2}{m} \Bigg[& -i\frac{2}{\tau}\mu \zeta_e^{-\mu}(1-\zeta_e)^{\nu} {}_2F_1(-\mu+\nu-\lambda, -\mu+\nu+\lambda; 1-2\mu; \zeta_e) \\ & - i\frac{2}{\tau} \zeta_e^{-\mu+1} \nu (1-\zeta_e)^{\nu-1} {}_2F_1(-\mu+\nu-\lambda, -\mu+\nu+\lambda; 1-2\mu; \zeta_e) \\ & + i\frac{2}{\tau} \zeta_e^{-\mu+1}(1-\zeta_e)^{\nu} \frac{(-\mu+\nu-\lambda)(-\mu+\nu+\lambda)}{1-2\mu} {}_2F_1(-\mu+\nu-\lambda+1, -\mu+\nu+\lambda+1; 1-2\mu+1; \zeta_e) \\ & - \left(p - \frac{qA_1 - qA_2\zeta_e}{1-\zeta_e}\right) \zeta_e^{-\mu}(1-\zeta_e)^{\nu} {}_2F_1(-\mu+\nu-\lambda, -\mu+\nu+\lambda; 1-2\mu; \zeta_e) \Bigg].\end{aligned} \tag{113}$$

7.3 Solution for the Later Medium ($t > t_0$)

For the later (l) medium, we use the variable

$$\zeta_l = -e^{-2\frac{t-t_0}{\tau}}, \tag{114}$$

which simplifies Eq. (100) to

$$A_z(\zeta_l) = \frac{A_2 - A_1\zeta_l}{1-\zeta_l}, \tag{115a}$$

and whose first and second time derivatives lead to

$$\frac{d}{dt} = -\frac{2}{\tau}\zeta_l \frac{d}{d\zeta_l} \tag{115b}$$

and

$$\frac{d^2}{dt^2} = \frac{4}{\tau^2}\zeta_l \frac{d}{d\zeta_l} + \frac{4}{\tau^2}\zeta_l^2 \frac{d^2}{d\zeta_l^2} \tag{115c}$$

respectively.

Inserting Eqs. (115) into Eq. (99) and multiplying the result by $\frac{\tau^2}{4}\frac{1-\zeta_1}{\zeta_1}$, we get, after some algebraic manipulations,

$$\zeta_1(1-\zeta_1)\frac{d^2\varphi_1}{d\zeta_1^2}+(1-\zeta_1)\frac{d\varphi_1}{d\zeta_1}+\frac{1}{\zeta_1(1-\zeta_1)}\left[\beta_1\zeta_1^2+\beta_0\zeta_1+\beta_2\right]\varphi_1=0, \tag{116a}$$

where

$$\beta_0=-\frac{\tau^2}{4}\left[((p-qA_2)^2+m^2)+((p-qA_1)^2+m^2)-(qA_2-qA_1)^2-2i\frac{qA_2-qA_1}{\tau}\right], \tag{116b}$$

$$\beta_1=\frac{\tau^2}{4}\left[(p-qA_1)^2+m^2\right]=\frac{\tau^2}{4}E_1^2, \tag{116c}$$

and

$$\beta_2=\frac{\tau^2}{4}\left[(p-qA_2)^2+m^2\right]=\frac{\tau^2}{4}E_2^2, \tag{116d}$$

and whose singularities at $\zeta_1=0$ and $1-\zeta_1=0$ can be removed by inserting the expression

$$\varphi_1(\zeta_1)=\zeta_1^{\sigma}(1-\zeta_1)^{\rho}f(\zeta_1), \tag{117}$$

its derivative,

$$\frac{d\varphi_1}{d\zeta_1}=\zeta_1^{\sigma}(1-\zeta_1)^{\rho}\left[\left(\sigma\frac{1}{\zeta_1}-\rho\frac{1}{1-\zeta_1}\right)f+\frac{df}{d\zeta_1}\right], \tag{118a}$$

and its second derivative,

$$\frac{d^2\varphi_1}{d\zeta_1^2}=\zeta_1^{\sigma}(1-\zeta_1)^{\rho}\left[\left(\sigma(\sigma-1)\frac{1}{\zeta_1^2}+\rho(\rho-1)\frac{1}{(1-\zeta_1)^2}-2\sigma\rho\frac{1}{\zeta_1(1-\zeta_1)}\right)f+\left(2\sigma\frac{1}{\zeta_1}-2\rho\frac{1}{1-\zeta_1}\right)\frac{df}{d\zeta_1}+\frac{d^2f}{d\zeta_1^2}\right]. \tag{118b}$$

Dividing the resulting equation by $\zeta_1^{\sigma}(1-\zeta_1)^{\rho}$ finally yields the hypergeometric-type equation

$$\zeta_1(1-\zeta_1)\frac{d^2f}{d\zeta_1^2}+\left[(2\sigma+1)-(2\sigma+2\rho+1)\zeta_1\right]\frac{df}{d\zeta_1}-\left[(\sigma+\rho+\eta)(\sigma+\rho-\eta)\right]f=0, \tag{119a}$$

where

$$\sigma=i\sqrt{\beta_2}=i\frac{\tau}{2}E_2, \tag{119b}$$

$$\rho=\frac{1-\sqrt{1-4(\beta_0+\beta_1+\beta_2)}}{2}=i\frac{\tau}{2}(qA_2-qA_1), \tag{119c}$$

and

$$\eta=i\sqrt{\beta_1}=i\frac{\tau}{2}E_1. \tag{119d}$$

The solution to Eqs. (119) can be expressed in terms of Gauss hypergeometric functions ${}_2F_1(\cdot)$[11] as

$$f(\zeta_1)=C_3\,{}_2F_1(\sigma+\rho-\eta,\sigma+\rho+\eta;1+2\sigma;\zeta_1)+C_4\zeta_1^{-2\sigma}\,{}_2F_1(-\sigma+\rho-\eta,-\sigma+\rho+\eta;1-2\sigma;\zeta_1). \tag{120}$$

and substituting this equation into Eq. (117) allows to determine the wavefunction $\varphi_1(\zeta_1)$ in terms of Gauss hypergeometric functions as

$$\begin{aligned}\varphi_1(\zeta_1)=&\,C_3\zeta_1^{\sigma}(1-\zeta_1)^{\rho}\,{}_2F_1(\sigma+\rho-\eta,\sigma+\rho+\eta;1+2\sigma;\zeta_1)\\&+C_4\zeta_1^{-\sigma}(1-\zeta_1)^{\rho}\,{}_2F_1(-\sigma+\rho-\eta,-\sigma+\rho+\eta;1-2\sigma;\zeta_1).\end{aligned} \tag{121}$$

To complete the spinor wavefunction, we also need to calculate ϑ_l. This may be accomplished by inserting Eq. (121) into Eq. (97) and using Eqs. (115a) and (115b). This includes the intermediate step

$$\begin{aligned}\frac{d\varphi_l}{d\zeta_l} &= C_3\sigma\zeta_l^{\sigma-1}(1-\zeta_l)^{\rho}{}_2F_1(\sigma+\rho-\eta,\sigma+\rho+\eta;1+2\sigma;\zeta_l)\\ &-C_3\zeta_l^{\sigma}\rho(1-\zeta_l)^{\rho-1}{}_2F_1(\sigma+\rho-\eta,\sigma+\rho+\eta;1+2\sigma;\zeta_l)\\ &+C_3\zeta_l^{\sigma}(1-\zeta_l)^{\rho}\frac{(\sigma+\rho-\eta)(\sigma+\rho+\eta)}{1-2\sigma}{}_2F_1(\sigma+\rho-\eta+1,\sigma+\rho+\eta+1;1+2\sigma+1;\zeta_l)\\ &-C_4\sigma\zeta_l^{-\sigma-1}(1-\zeta_l)^{\rho}{}_2F_1(-\sigma+\rho-\eta,-\sigma+\rho+\eta;1-2\sigma;\zeta_l)\\ &-C_4\zeta_l^{-\sigma}\rho(1-\zeta_l)^{\rho-1}{}_2F_1(-\sigma+\rho-\eta,-\sigma+\rho+\eta;1-2\sigma;\zeta_l)\\ &+C_4\zeta_l^{-\sigma}(1-\zeta_l)^{\rho}\frac{(-\sigma+\rho-\eta)(-\sigma+\rho+\eta)}{1-2\sigma}{}_2F_1(-\sigma+\rho-\eta+1,-\sigma+\rho+\eta+1;1-2\sigma+1;\zeta_l),\end{aligned} \tag{122}$$

where using the derivative property of hypergeometric functions given in Eq. (112) leads to

$$\begin{aligned}\vartheta_l = \frac{C_3}{m}\Bigg[&-i\frac{2}{\tau}\sigma\zeta_l^{\sigma}(1-\zeta_l)^{\rho}{}_2F_1(\sigma+\rho-\eta,\sigma+\rho+\eta;1+2\sigma;\zeta_l)\\ &+i\frac{2}{\tau}\zeta_l^{\sigma+1}\rho(1-\zeta_l)^{\rho-1}{}_2F_1(\sigma+\rho-\eta,\sigma+\rho+\eta;1+2\sigma;\zeta_l)\\ &-i\frac{2}{\tau}\zeta_l^{\sigma+1}(1-\zeta_l)^{\rho}\frac{(\sigma+\rho-\eta)(\sigma+\rho+\eta)}{1-2\sigma}{}_2F_1(\sigma+\rho-\eta+1,\sigma+\rho+\eta+1;1+2\sigma+1;\zeta_l)\\ &-\left(p-\frac{qA_2-qA_1\zeta_l}{1-\zeta_l}\right)\zeta_l^{\sigma}(1-\zeta_l)^{\rho}{}_2F_1(\sigma+\rho-\eta,\sigma+\rho+\eta;1+2\sigma;\zeta_l)\Bigg]\\ +\frac{C_4}{m}\Bigg[&i\frac{2}{\tau}\sigma\zeta_l^{-\sigma}(1-\zeta_l)^{\rho}{}_2F_1(-\sigma+\rho-\eta,-\sigma+\rho+\eta;1-2\sigma;\zeta_l)\\ &+i\frac{2}{\tau}\zeta_l^{-\sigma+1}\rho(1-\zeta_l)^{\rho-1}{}_2F_1(-\sigma+\rho-\eta,-\sigma+\rho+\eta;1-2\sigma;\zeta_l)\\ &-i\frac{2}{\tau}\zeta_l^{-\sigma+1}(1-\zeta_l)^{\rho}\frac{(-\sigma+\rho-\eta)(-\sigma+\rho+\eta)}{1-2\sigma}{}_2F_1(-\sigma+\rho-\eta+1,-\sigma+\rho+\eta+1;1-2\sigma+1;\zeta_l)\\ &-\left(p-\frac{qA_2-qA_1\zeta_l}{1-\zeta_l}\right)\zeta_l^{-\sigma}(1-\zeta_l)^{\rho}{}_2F_1(-\sigma+\rho-\eta,-\sigma+\rho+\eta;1-2\sigma;\zeta_l)\Bigg].\end{aligned} \tag{123}$$

7.4 Asymptotic Forms

Being fundamentally different from its infinitely sharp counterpart in the vicinity of its transition, the smooth potential [Fig. 3(a)] can obviously not be compared with the infinitely sharp potential in that region. Instead, the comparison must be carried out sufficiently far (in time) from the transition, and we should therefore resort to an asymptotic evaluation of the above exact solutions for the smooth potential step.

7.4.1 Asymptotic Forms for the Earlier Medium ($t < t_0$)

The asymptotic regime for the earlier medium corresponds to the limit $t \to -\infty$, where $\zeta_e \to 0$ and $(1-\zeta_e) \to 1$, and $F(a,b;c;\zeta_e) \to 1$[11]. Inserting these limits into Eq. (109) yields the asymptotic form of φ_e in terms of ζ_e,

$$\varphi_e(\zeta_e \to 0) = C_1\zeta_e^{\mu} + C_2\zeta_e^{-\mu}, \tag{124}$$

and subsequently in terms of t

$$\varphi_e(t \to -\infty) = C_1(-e^{2\frac{t-t_0}{\tau}})^{i\sqrt{\alpha_1}} + C_2(-e^{2\frac{t-t_0}{\tau}})^{-i\sqrt{\alpha_1}}, \tag{125}$$

while inserting Eq. (125) and Eq. (100) into Eq. (97) yields

$$\vartheta_e(t \to -\infty) = \frac{C_1}{m}\left[-\frac{2}{\tau}\sqrt{\alpha_1}-(p-qA_1)\right](-e^{2\frac{t-t_0}{\tau}})^{i\sqrt{\alpha_1}} + \frac{C_2}{m}\left[\frac{2}{\tau}\sqrt{\alpha_1}-(p-qA_1)\right](-e^{2\frac{t-t_0}{\tau}})^{-i\sqrt{\alpha_1}}. \tag{126}$$

Finally, since there is only the incident wave in the earlier medium, the coefficient C_1 must be zero, which reduces Eq. (125) and (126) to

$$\varphi_e(t \to -\infty) = C_2(-e^{2\frac{t-t_0}{\tau}})^{-i\sqrt{\alpha_1}} \tag{127a}$$

and

$$\vartheta_{\mathrm{e}}(t\to-\infty)=\frac{C_2}{m}\left[\frac{2}{\tau}\sqrt{\alpha_1}-(p-qA_1)\right](-\mathrm{e}^{2\frac{t-t_0}{\tau}})^{-i\sqrt{\alpha_1}}. \tag{127b}$$

7.4.2 Asymptotic Forms for the Later Medium ($t>t_0$)

The asymptotic regime for the later medium corresponds to the limit $t\to\infty$, where $\zeta_1\to0$ and $(1-\zeta_{\mathrm{e}})\to1$, and $F(a,b;c;\zeta_1)\to1$[11]. Inserting these limits into Eq. (121) yields the asymptotic form of φ_1 in terms of ζ_1,

$$\varphi_1(\zeta_1\to0)=C_3\zeta_1^{\sigma}+C_4\zeta_1^{-\sigma}, \tag{128}$$

and subsequently in terms of t

$$\varphi_1(t\to+\infty)=C_3(-\mathrm{e}^{-2\frac{t-t_0}{\tau}})^{i\sqrt{\beta_2}}+C_4(-\mathrm{e}^{-2\frac{t-t_0}{\tau}})^{-i\sqrt{\beta_2}}, \tag{129}$$

while inserting Eq. (129) and Eq. (100) into Eq. (97) yields

$$\vartheta_1(t\to+\infty)=\frac{C_3}{m}\left[\frac{2}{\tau}\sqrt{\beta_2}-(p-qA_2)\right](-\mathrm{e}^{-2\frac{t-t_0}{\tau}})^{i\sqrt{\beta_2}}+\frac{C_4}{m}\left[-\frac{2}{\tau}\sqrt{\beta_2}-(p-qA_2)\right](-\mathrm{e}^{-2\frac{t-t_0}{\tau}})^{-i\sqrt{\beta_2}}. \tag{130}$$

7.5 Weyl to Dirac Spinor Transformation

For consistency with the mathematical forms of the spinors used throughout this paper, we transform our spinors from the Weyl representation to the Dirac representation. This ensures that our calculations remain consistent and comparable with the results established in the paper. This transformation[12] may be written as

$$\psi(\mathrm{Dirac})=U\psi(\mathrm{Weyl}),\quad \text{where}\quad U=\frac{1}{\sqrt{2}}\begin{pmatrix}1&1\\1&-1\end{pmatrix}. \tag{131}$$

If we denote the Dirac and Weyl spinors as

$$\psi(\mathrm{Dirac})=\begin{pmatrix}\varphi^{\mathrm{D}}\\ \vartheta^{\mathrm{D}}\end{pmatrix}\quad\text{and}\quad \psi(\mathrm{Weyl})=\begin{pmatrix}\varphi\\ \vartheta\end{pmatrix}, \tag{132}$$

we find using Eq. (131) that the spinor in the Dirac representation in terms of the elements of the spinor in the Weyl representation read

$$\begin{pmatrix}\varphi^{\mathrm{D}}\\ \vartheta^{\mathrm{D}}\end{pmatrix}=\frac{1}{\sqrt{2}}\begin{pmatrix}\varphi+\vartheta\\ \varphi-\vartheta\end{pmatrix}. \tag{133}$$

For the earlier medium, inserting Eq. (127a) and Eq. (127b) into the first row of Eq. (133), we find

$$\varphi_{\mathrm{e}}^{\mathrm{D}}(t\to-\infty)=\frac{1}{\sqrt{2}}\left(C_2(-\mathrm{e}^{2\frac{t-t_0}{\tau}})^{-i\sqrt{\alpha_1}}+\frac{C_2}{m}\left[\frac{2}{\tau}\sqrt{\alpha_1}-(p-qA_1)\right](-\mathrm{e}^{2\frac{t-t_0}{\tau}})^{-i\sqrt{\alpha_1}}\right), \tag{134}$$

which, upon substituting Eq. (104c) and using $(-1)^a=\mathrm{e}^{i\pi a}$, transforms to the the asymptotic form

$$\varphi_{\mathrm{e}}^{\mathrm{D}}(t\to-\infty)=\frac{1}{\sqrt{2}}\frac{C_2}{m}\mathrm{e}^{\pi\frac{\tau}{2}E_1}\left(m+E_1-(p-qA_1)\right)\mathrm{e}^{-iE_1(t-t_0)}. \tag{135}$$

Similarly, inserting Eq. (127a) and Eq. (127b) into the second row of Eq. (133), then substituting Eq. (104c) into the resulting expression and using $(-1)^a=\mathrm{e}^{i\pi a}$, we find

$$\vartheta_{\mathrm{e}}^{\mathrm{D}}(t\to-\infty)=\frac{1}{\sqrt{2}}\frac{C_2}{m}\mathrm{e}^{\pi\frac{\tau}{2}E_1}\left(m-E_1+(p-qA_1)\right)\mathrm{e}^{-iE_1(t-t_0)}. \tag{136}$$

This equation may be algebraically manipulated so as to take a form including an expression that resembles the wave function for the infinitely sharp step [Eq. (49a)], viz.,

$$\vartheta_{\mathrm{e}}^{\mathrm{D}}(t\to-\infty)=\frac{1}{\sqrt{2}}\frac{C_2}{m}\mathrm{e}^{\pi\frac{\tau}{2}E_1}\left(m+E_1-(p-qA_1)\right)\left(\frac{E_1-m}{p-qA_1}\right)\mathrm{e}^{-iE_1(t-t_0)}. \tag{137}$$

Thus, the Dirac spinor wavefunction for the earlier medium is

$$\psi_{\rm e}^{\rm D}(t\to-\infty,z)=\begin{pmatrix}\varphi_{\rm e}^{\rm D}(t\to-\infty)\\ \vartheta_{\rm e}^{\rm D}(t\to-\infty)\end{pmatrix}e^{ipz}=\frac{1}{\sqrt{2}}\frac{C_2}{m}{\rm e}^{\pi\frac{\tau}{2}E_1}\left(m+E_1-(p-qA_1)\right)\begin{pmatrix}1\\ \frac{E_1-m}{p-qA_1}\end{pmatrix}{\rm e}^{-iE_1(t-t_0)}e^{ipz}. \tag{138}$$

For the later medium, inserting Eq. (129) and Eq. (130) into the first row of Eq. (133), we find

$$\begin{aligned}\varphi_{\rm l}^{\rm D}(t\to+\infty)=\frac{1}{\sqrt{2}}\Big(&C_3(-{\rm e}^{-2\frac{t-t_0}{\tau}})^{i\sqrt{\beta_2}}+C_4(-{\rm e}^{-2\frac{t-t_0}{\tau}})^{-i\sqrt{\beta_2}}\\&+\frac{C_3}{m}\left[\frac{2}{\tau}\sqrt{\beta_2}-(p-qA_2)\right](-{\rm e}^{-2\frac{t-t_0}{\tau}})^{i\sqrt{\beta_2}}+\frac{C_4}{m}\left[-\frac{2}{\tau}\sqrt{\beta_2}-(p-qA_2)\right](-{\rm e}^{-2\frac{t-t_0}{\tau}})^{-i\sqrt{\beta_2}}\Big),\end{aligned} \tag{139}$$

which, upon substituting Eq. (116d) and using $(-1)^a={\rm e}^{i\pi a}$, transforms to the the asymptotic form

$$\begin{aligned}\varphi_{\rm l}^{\rm D}(t\to+\infty)=&\frac{1}{\sqrt{2}}\frac{C_3}{m}{\rm e}^{-\pi\frac{\tau}{2}E_2}\left(m+E_2-(p-qA_2)\right){\rm e}^{-iE_2(t-t_0)}\\&+\frac{1}{\sqrt{2}}\frac{C_4}{m}{\rm e}^{\pi\frac{\tau}{2}E_2}\left(m-E_2-(p-qA_2)\right){\rm e}^{iE_2(t-t_0)}.\end{aligned} \tag{140}$$

Similarly, inserting Eq. (129) and Eq. (130) into the second row of Eq. (133), then substituting Eq. (104c) into the resulting expression and using $(-1)^a={\rm e}^{i\pi a}$, we find

$$\begin{aligned}\vartheta_{\rm l}^{\rm D}(t\to+\infty)=&\frac{1}{\sqrt{2}}\frac{C_3}{m}{\rm e}^{-\pi\frac{\tau}{2}E_2}\left(m-E_2+(p-qA_2)\right){\rm e}^{-iE_2(t-t_0)}\\&+\frac{1}{\sqrt{2}}\frac{C_4}{m}{\rm e}^{\pi\frac{\tau}{2}E_2}\left(m+E_2+(p-qA_2)\right){\rm e}^{iE_2(t-t_0)}.\end{aligned} \tag{141}$$

This equation may be algebraically manipulated so as to take a form including an expression that resembles the wave function for the infinitely sharp step [Eq. (49b)], viz.,

$$\begin{aligned}\vartheta_{\rm l}^{\rm D}(t\to+\infty)=&\frac{1}{\sqrt{2}}\frac{C_3}{m}{\rm e}^{-\pi\frac{\tau}{2}E_2}\left(m+E_2-(p-qA_2)\right)\left(\frac{E_2-m}{p-qA_2}\right){\rm e}^{-iE_2(t-t_0)}\\&+\frac{1}{\sqrt{2}}\frac{C_4}{m}{\rm e}^{\pi\frac{\tau}{2}E_2}\left(m-E_2-(p-qA_2)\right)\left(\frac{-E_2-m}{p-qA_2}\right){\rm e}^{iE_2(t-t_0)},\end{aligned} \tag{142}$$

Thus, the Dirac spinor wavefunction for the later medium is

$$\begin{aligned}\psi_{\rm l}^{\rm D}(t\to+\infty,z)=\begin{pmatrix}\varphi_{\rm l}^{\rm D}(t\to+\infty)\\ \vartheta_{\rm l}^{\rm D}(t\to+\infty)\end{pmatrix}e^{ipz}=&\frac{1}{\sqrt{2}}\frac{C_3}{m}{\rm e}^{-\pi\frac{\tau}{2}E_2}\left(m+E_2-(p-qA_2)\right)\begin{pmatrix}1\\ \frac{E_2-m}{p-qA_2}\end{pmatrix}{\rm e}^{-iE_2(t-t_0)}e^{ipz}\\&+\frac{1}{\sqrt{2}}\frac{C_4}{m}{\rm e}^{\pi\frac{\tau}{2}E_2}\left(m-E_2-(p-qA_2)\right)\begin{pmatrix}1\\ \frac{-E_2-m}{p-qA_2}\end{pmatrix}{\rm e}^{iE_2(t-t_0)}e^{ipz}.\end{aligned} \tag{143}$$

Inserting Eq. (110) and Eq. (113) into the first row of Eq. (133), we find the *general form* of $\varphi_{\rm e}$ in the Dirac representation as

$$\begin{aligned}\varphi_{\rm e}^{\rm D}=\frac{1}{\sqrt{2}}\frac{C_2}{m}\Big[&m\zeta_{\rm e}^{-\mu}(1-\zeta_{\rm e})^{\nu}{}_2F_1(-\mu+\nu-\lambda,-\mu+\nu+\lambda;1-2\mu;\zeta_{\rm e})\\&-i\frac{2}{\tau}\mu\zeta_{\rm e}^{-\mu}(1-\zeta_{\rm e})^{\nu}{}_2F_1(-\mu+\nu-\lambda,-\mu+\nu+\lambda;1-2\mu;\zeta_{\rm e})\\&-i\frac{2}{\tau}\zeta_{\rm e}^{-\mu+1}\nu(1-\zeta_{\rm e})^{\nu-1}{}_2F_1(-\mu+\nu-\lambda,-\mu+\nu+\lambda;1-2\mu;\zeta_{\rm e})\\&+i\frac{2}{\tau}\zeta_{\rm e}^{-\mu+1}(1-\zeta_{\rm e})^{\nu}\frac{(-\mu+\nu-\lambda)(-\mu+\nu+\lambda)}{1-2\mu}{}_2F_1(-\mu+\nu-\lambda+1,-\mu+\nu+\lambda+1;1-2\mu+1;\zeta_{\rm e})\\&-\left(p-\frac{qA_1-qA_2\zeta_{\rm e}}{1-\zeta_{\rm e}}\right)\zeta_{\rm e}^{-\mu}(1-\zeta_{\rm e})^{\nu}{}_2F_1(-\mu+\nu-\lambda,-\mu+\nu+\lambda;1-2\mu;\zeta_{\rm e})\Big].\end{aligned} \tag{144}$$

Similarly, inserting Eq. (110) and Eq. (113) into the second row of Eq. (133), we find the general form of $\vartheta_{\rm e}$ in the Dirac representation as

$$\begin{aligned}
\vartheta_{\rm e}^{\rm D} = \frac{1}{\sqrt{2}}\frac{C_2}{m}\Big[& m\zeta_{\rm e}^{-\mu}(1-\zeta_{\rm e})^{\nu}{}_2F_1(-\mu+\nu-\lambda,-\mu+\nu+\lambda;1-2\mu;\zeta_{\rm e}) \\
& +i\frac{2}{\tau}\mu\zeta_{\rm e}^{-\mu}(1-\zeta_{\rm e})^{\nu}{}_2F_1(-\mu+\nu-\lambda,-\mu+\nu+\lambda;1-2\mu;\zeta_{\rm e}) \\
& +i\frac{2}{\tau}\zeta_{\rm e}^{-\mu+1}\nu(1-\zeta_{\rm e})^{\nu-1}{}_2F_1(-\mu+\nu-\lambda,-\mu+\nu+\lambda;1-2\mu;\zeta_{\rm e}) \\
& -i\frac{2}{\tau}\zeta_{\rm e}^{-\mu+1}(1-\zeta_{\rm e})^{\nu}\frac{(-\mu+\nu-\lambda)(-\mu+\nu+\lambda)}{1-2\mu}{}_2F_1(-\mu+\nu-\lambda+1,-\mu+\nu+\lambda+1;1-2\mu+1;\zeta_{\rm e}) \\
& +\left(p-\frac{qA_1-qA_2\zeta_{\rm e}}{1-\zeta_{\rm e}}\right)\zeta_{\rm e}^{-\mu}(1-\zeta_{\rm e})^{\nu}{}_2F_1(-\mu+\nu-\lambda,-\mu+\nu+\lambda;1-2\mu;\zeta_{\rm e})\Big].
\end{aligned} \tag{145}$$

Still similarly, inserting Eq. (121) and Eq. (123) into the first row of Eq. (133), we find the general form of $\varphi_{\rm l}$ in the Dirac representation as

$$\begin{aligned}
\varphi_{\rm l}^{\rm D} = \frac{1}{\sqrt{2}}\frac{C_3}{m}\Big[& m\zeta_{\rm l}^{\sigma}(1-\zeta_{\rm l})^{\rho}{}_2F_1(\sigma+\rho-\eta,\sigma+\rho+\eta;1+2\sigma;\zeta_{\rm l}) \\
& -i\frac{2}{\tau}\sigma\zeta_{\rm l}^{\sigma}(1-\zeta_{\rm l})^{\rho}{}_2F_1(\sigma+\rho-\eta,\sigma+\rho+\eta;1+2\sigma;\zeta_{\rm l}) \\
& +i\frac{2}{\tau}\zeta_{\rm l}^{\sigma+1}\rho(1-\zeta_{\rm l})^{\rho-1}{}_2F_1(\sigma+\rho-\eta,\sigma+\rho+\eta;1+2\sigma;\zeta_{\rm l}) \\
& -i\frac{2}{\tau}\zeta_{\rm l}^{\sigma+1}(1-\zeta_{\rm l})^{\rho}\frac{(\sigma+\rho-\eta)(\sigma+\rho+\eta)}{1-2\sigma}{}_2F_1(\sigma+\rho-\eta+1,\sigma+\rho+\eta+1;1+2\sigma+1;\zeta_{\rm l}) \\
& -\left(p-\frac{qA_2-qA_1\zeta_{\rm l}}{1-\zeta_{\rm l}}\right)\zeta_{\rm l}^{\sigma}(1-\zeta_{\rm l})^{\rho}{}_2F_1(\sigma+\rho-\eta,\sigma+\rho+\eta;1+2\sigma;\zeta_{\rm l})\Big] \\
+\frac{1}{\sqrt{2}}\frac{C_4}{m}\Big[& m\zeta_{\rm l}^{-\sigma}(1-\zeta_{\rm l})^{\rho}{}_2F_1(-\sigma+\rho-\eta,-\sigma+\rho+\eta;1-2\sigma;\zeta_{\rm l}) \\
& +i\frac{2}{\tau}\sigma\zeta_{\rm l}^{-\sigma}(1-\zeta_{\rm l})^{\rho}{}_2F_1(-\sigma+\rho-\eta,-\sigma+\rho+\eta;1-2\sigma;\zeta_{\rm l}) \\
& +i\frac{2}{\tau}\zeta_{\rm l}^{-\sigma+1}\rho(1-\zeta_{\rm l})^{\rho-1}{}_2F_1(-\sigma+\rho-\eta,-\sigma+\rho+\eta;1-2\sigma;\zeta_{\rm l}) \\
& -i\frac{2}{\tau}\zeta_{\rm l}^{-\sigma+1}(1-\zeta_{\rm l})^{\rho}\frac{(-\sigma+\rho-\eta)(-\sigma+\rho+\eta)}{1-2\sigma}{}_2F_1(-\sigma+\rho-\eta+1,-\sigma+\rho+\eta+1;1-2\sigma+1;\zeta_{\rm l}) \\
& -\left(p-\frac{qA_2-qA_1\zeta_{\rm l}}{1-\zeta_{\rm l}}\right)\zeta_{\rm l}^{-\sigma}(1-\zeta_{\rm l})^{\rho}{}_2F_1(-\sigma+\rho-\eta,-\sigma+\rho+\eta;1-2\sigma;\zeta_{\rm l})\Big],
\end{aligned} \tag{146}$$

and finally inserting Eq. (121) and Eq. (123) into the second row of Eq. (133), we find the general form of $\vartheta_{\rm l}$ in the Dirac

representation as

$$\vartheta_{l}^{D}=\frac{1}{\sqrt{2}}\frac{C_3}{m}\Big[m\zeta_l^{\sigma}(1-\zeta_l)^{\rho}{}_2F_1(\sigma+\rho-\eta,\sigma+\rho+\eta;1+2\sigma;\zeta_l)$$
$$+i\frac{2}{\tau}\sigma\zeta_l^{\sigma}(1-\zeta_l)^{\rho}{}_2F_1(\sigma+\rho-\eta,\sigma+\rho+\eta;1+2\sigma;\zeta_l)$$
$$-i\frac{2}{\tau}\zeta_l^{\sigma+1}\rho(1-\zeta_l)^{\rho-1}{}_2F_1(\sigma+\rho-\eta,\sigma+\rho+\eta;1+2\sigma;\zeta_l)$$
$$+i\frac{2}{\tau}\zeta_l^{\sigma+1}(1-\zeta_l)^{\rho}\frac{(\sigma+\rho-\eta)(\sigma+\rho+\eta)}{1-2\sigma}{}_2F_1(\sigma+\rho-\eta+1,\sigma+\rho+\eta+1;1+2\sigma+1;\zeta_l)$$
$$+\left(p-\frac{qA_2-qA_1\zeta_l}{1-\zeta_l}\right)\zeta_l^{\sigma}(1-\zeta_l)^{\rho}{}_2F_1(\sigma+\rho-\eta,\sigma+\rho+\eta;1+2\sigma;\zeta_l)\Big]$$
$$+\frac{1}{\sqrt{2}}\frac{C_4}{m}\Big[m\zeta_l^{-\sigma}(1-\zeta_l)^{\rho}{}_2F_1(-\sigma+\rho-\eta,-\sigma+\rho+\eta;1-2\sigma;\zeta_l)$$
$$-i\frac{2}{\tau}\sigma\zeta_l^{-\sigma}(1-\zeta_l)^{\rho}{}_2F_1(-\sigma+\rho-\eta,-\sigma+\rho+\eta;1-2\sigma;\zeta_l)$$
$$-i\frac{2}{\tau}\zeta_l^{-\sigma+1}\rho(1-\zeta_l)^{\rho-1}{}_2F_1(-\sigma+\rho-\eta,-\sigma+\rho+\eta;1-2\sigma;\zeta_l)$$
$$+i\frac{2}{\tau}\zeta_l^{-\sigma+1}(1-\zeta_l)^{\rho}\frac{(-\sigma+\rho-\eta)(-\sigma+\rho+\eta)}{1-2\sigma}{}_2F_1(-\sigma+\rho-\eta+1,-\sigma+\rho+\eta+1;1-2\sigma+1;\zeta_l)$$
$$+\left(p-\frac{qA_2-qA_1\zeta_l}{1-\zeta_l}\right)\zeta_l^{-\sigma}(1-\zeta_l)^{\rho}{}_2F_1(-\sigma+\rho-\eta,-\sigma+\rho+\eta;1-2\sigma;\zeta_l)\Big]. \tag{147}$$

7.6 Boundary Conditions

At this point, we need to determine the coefficient ratios C_3/C_2 and C_4/C_2 to be able to calculate probability coefficients. For this purpose, we apply the boundary conditions to the wavefunctions in the general forms. Let us first write the Dirac wavefunctions in (Eqs. (144), (146), (145), and (147)) at $t=t_0$, which will imply the use of the following relations:

- at $t=t_0$, $\zeta_e=\zeta_l=-1$ since $\zeta_e=-e^{2\frac{t-t_0}{\tau}}$ and $\zeta_l=-e^{-2\frac{t-t_0}{\tau}}$,
- $(-1)^a=e^{i\pi a}$ and $e^{i\pi}=-1$,
- $\rho=\nu$, $\eta=\mu$, and $\sigma=\lambda$, according to Eqs. (107b), (107c), (107d), (119b), (119c) and (119d).

At $t=t_0$, the wavefunctions in Eqs. (144), (145), (146), and (147) may be written as

$$\varphi_e^{D}(t=t_0)=\frac{2^{\nu-1}}{\sqrt{2}}\frac{C_2}{m}e^{\pi\frac{\tau}{2}E_1}\left[(2m+\mathscr{D}_1)\mathscr{F}_1-\mathscr{D}_2\mathscr{F}_2\right], \tag{148a}$$

$$\varphi_l^{D}(t=t_0)=\frac{2^{\nu-1}}{\sqrt{2}}\frac{C_3}{m}e^{-\pi\frac{\tau}{2}E_2}\left[(2m+\mathscr{D}_3)\mathscr{F}_3+\mathscr{D}_4\mathscr{F}_4\right]+\frac{2^{\nu-1}}{\sqrt{2}}\frac{C_4}{m}e^{\pi\frac{\tau}{2}E_2}\left[(2m-\mathscr{D}_5)\mathscr{F}_5+\mathscr{D}_6\mathscr{F}_6\right], \tag{148b}$$

$$\vartheta_e^{D}(t=t_0)=\frac{2^{\nu-1}}{\sqrt{2}}\frac{C_2}{m}e^{\pi\frac{\tau}{2}E_1}\left[(2m-\mathscr{D}_1)\mathscr{F}_1+\mathscr{D}_2\mathscr{F}_2\right], \tag{148c}$$

$$\vartheta_l^{D}(t=t_0)=\frac{2^{\nu-1}}{\sqrt{2}}\frac{C_3}{m}e^{-\pi\frac{\tau}{2}E_2}\left[(2m-\mathscr{D}_3)\mathscr{F}_3-\mathscr{D}_4\mathscr{F}_4\right]+\frac{2^{\nu-1}}{\sqrt{2}}\frac{C_4}{m}e^{\pi\frac{\tau}{2}E_2}\left[(2m+\mathscr{D}_5)\mathscr{F}_5-\mathscr{D}_6\mathscr{F}_6\right], \tag{148d}$$

where

$$\mathscr{F}_1={}_2F_1(-\mu+\nu-\lambda,-\mu+\nu+\lambda;1-2\mu;-1), \tag{149a}$$

$$\mathscr{F}_2={}_2F_1(-\mu+\nu-\lambda+1,-\mu+\nu+\lambda+1;1-2\mu+1;-1), \tag{149b}$$

$$\mathscr{F}_3 = {}_2F_1(\lambda+\nu-\mu, \lambda+\nu+\mu; 1+2\lambda; -1), \tag{149c}$$

$$\mathscr{F}_4 = {}_2F_1(\lambda+\nu-\mu+1, \lambda+\nu+\mu+1; 1+2\lambda+1; -1), \tag{149d}$$

$$\mathscr{F}_5 = {}_2F_1(-\lambda+\nu-\mu, -\lambda+\nu+\mu; 1-2\lambda; -1), \tag{149e}$$

$$\mathscr{F}_6 = {}_2F_1(-\lambda+\nu-\mu+1, -\lambda+\nu+\mu+1; 1-2\lambda+1; -1) \tag{149f}$$

and

$$\mathscr{D}_1 = 2E_1 - (qA_2 - qA_1) - (p - qA_2) - (p - qA_1), \tag{150a}$$

$$\mathscr{D}_2 = i\frac{4}{\tau}\frac{(-\mu+\nu-\lambda)(-\mu+\nu+\lambda)}{1-2\mu}, \tag{150b}$$

$$\mathscr{D}_3 = 2E_2 + (qA_2 - qA_1) - (p - qA_2) - (p - qA_1), \tag{150c}$$

$$\mathscr{D}_4 = i\frac{4}{\tau}\frac{(\lambda+\nu-\mu)(\lambda+\nu+\mu)}{1-2\lambda}, \tag{150d}$$

$$\mathscr{D}_5 = 2E_2 - (qA_2 - qA_1) + (p - qA_2) + (p - qA_1), \tag{150e}$$

$$\mathscr{D}_6 = i\frac{4}{\tau}\frac{(-\lambda+\nu-\mu)(-\lambda+\nu+\mu)}{1-2\lambda}. \tag{150f}$$

We can now apply the boundary conditions to the wave functions (148a) to (148b), and (148c) to (148d) as

$$\varphi_{\mathrm{e}}^{\mathrm{D}}(t=t_0) = \varphi_{\mathrm{l}}^{\mathrm{D}}(t=t_0) \tag{151a}$$

and

$$\vartheta_{\mathrm{e}}^{\mathrm{D}}(t=t_0) = \vartheta_{\mathrm{l}}^{\mathrm{D}}(t=t_0), \tag{151b}$$

which yields

$$\begin{aligned} \mathrm{e}^{\pi\frac{\tau}{2}E_1}\left[(2m+\mathscr{D}_1)\mathscr{F}_1 - \mathscr{D}_2\mathscr{F}_2\right] = {} & \frac{C_3}{C_2}\mathrm{e}^{-\pi\frac{\tau}{2}E_2}\left[(2m+\mathscr{D}_3)\mathscr{F}_3 + \mathscr{D}_4\mathscr{F}_4\right] \\ & + \frac{C_4}{C_2}\mathrm{e}^{\pi\frac{\tau}{2}E_2}\left[(2m-\mathscr{D}_5)\mathscr{F}_5 + \mathscr{D}_6\mathscr{F}_6\right] \end{aligned} \tag{152a}$$

and

$$\begin{aligned} \mathrm{e}^{\pi\frac{\tau}{2}E_1}\left[(2m-\mathscr{D}_1)\mathscr{F}_1 + \mathscr{D}_2\mathscr{F}_2\right] = {} & \frac{C_3}{C_2}\mathrm{e}^{-\pi\frac{\tau}{2}E_2}\left[(2m-\mathscr{D}_3)\mathscr{F}_3 - \mathscr{D}_4\mathscr{F}_4\right] \\ & + \frac{C_4}{C_2}\mathrm{e}^{\pi\frac{\tau}{2}E_2}\left[(2m+\mathscr{D}_5)\mathscr{F}_5 - \mathscr{D}_6\mathscr{F}_6\right], \end{aligned} \tag{152b}$$

which resolve to

$$\frac{C_3}{C_2} = \mathrm{e}^{\pi\frac{\tau}{2}(E_1+E_2)} \frac{[(2m-\mathscr{D}_1)\mathscr{F}_1+\mathscr{D}_2\mathscr{F}_2][(2m-\mathscr{D}_5)\mathscr{F}_5+\mathscr{D}_6\mathscr{F}_6]-[(2m+\mathscr{D}_1)\mathscr{F}_1-\mathscr{D}_2\mathscr{F}_2][(2m+\mathscr{D}_5)\mathscr{F}_5-\mathscr{D}_6\mathscr{F}_6]}{[(2m-\mathscr{D}_3)\mathscr{F}_3-\mathscr{D}_4\mathscr{F}_4][(2m-\mathscr{D}_5)\mathscr{F}_5+\mathscr{D}_6\mathscr{F}_6]-[(2m+\mathscr{D}_3)\mathscr{F}_3+\mathscr{D}_4\mathscr{F}_4][(2m+\mathscr{D}_5)\mathscr{F}_5-\mathscr{D}_6\mathscr{F}_6]} \tag{153a}$$

and

$$\frac{C_4}{C_2} = \mathrm{e}^{\pi\frac{\tau}{2}(E_1-E_2)} \frac{[(2m+\mathscr{D}_1)\mathscr{F}_1-\mathscr{D}_2\mathscr{F}_2][(2m-\mathscr{D}_3)\mathscr{F}_3-\mathscr{D}_4\mathscr{F}_4]-[(2m-\mathscr{D}_1)\mathscr{F}_1+\mathscr{D}_2\mathscr{F}_2][(2m+\mathscr{D}_3)\mathscr{F}_3+\mathscr{D}_4\mathscr{F}_4]}{[(2m-\mathscr{D}_3)\mathscr{F}_3-\mathscr{D}_4\mathscr{F}_4][(2m-\mathscr{D}_5)\mathscr{F}_5+\mathscr{D}_6\mathscr{F}_6]-[(2m+\mathscr{D}_3)\mathscr{F}_3+\mathscr{D}_4\mathscr{F}_4][(2m+\mathscr{D}_5)\mathscr{F}_5-\mathscr{D}_6\mathscr{F}_6]}. \tag{153b}$$

7.7 Asymptotic Spinor Wavefunctions and Scattering Probabilities

We shall finally calculate here the sought after scattering probabilities. For this purpose, we first recall the asymptotic Dirac spinors in Eqs. (138) and (143):

$$\psi_{\mathrm{e}}^{\mathrm{D}}(t\to-\infty,z) = G_{\mathrm{i}}\begin{pmatrix}1\\ \frac{E_1-m}{p-qA_1}\end{pmatrix}\mathrm{e}^{-iE_1(t-t_0)}e^{ipz} \tag{154a}$$

and

$$\psi_{\mathrm{l}}^{\mathrm{D}}(t\to+\infty,z) = G_{\mathrm{f}}\begin{pmatrix}1\\ \frac{E_2-m}{p-qA_2}\end{pmatrix}\mathrm{e}^{-iE_2(t-t_0)}e^{ipz} + G_{\mathrm{b}}\begin{pmatrix}1\\ \frac{-E_2-m}{p-qA_2}\end{pmatrix}\mathrm{e}^{iE_2(t-t_0)}e^{ipz}, \tag{154b}$$

where

$$G_{\mathrm{i}} = \frac{1}{\sqrt{2}}\frac{C_2}{m}\mathrm{e}^{\pi\frac{\tau}{2}E_1}\left(m+E_1-(p-qA_1)\right), \tag{154c}$$

$$G_{\mathrm{f}} = \frac{1}{\sqrt{2}}\frac{C_3}{m}\mathrm{e}^{-\pi\frac{\tau}{2}E_2}\left(m+E_2-(p-qA_2)\right), \tag{154d}$$

and

$$G_{\mathrm{b}} = \frac{1}{\sqrt{2}}\frac{C_4}{m}\mathrm{e}^{\pi\frac{\tau}{2}E_2}\left(m-E_2-(p-qA_2)\right). \tag{154e}$$

The later-forward and later-backward scattering coefficients may then directly be obtained from Eqs. (154c), (154d) and (154e) according to their definitions, as

$$f = \frac{G_{\mathrm{f}}}{G_{\mathrm{i}}} = \frac{\frac{1}{\sqrt{2}}\frac{C_3}{m}\mathrm{e}^{-\pi\frac{\tau}{2}E_2}\left(m+E_2-(p-qA_2)\right)}{\frac{1}{\sqrt{2}}\frac{C_2}{m}\mathrm{e}^{\pi\frac{\tau}{2}E_1}\left(m+E_1-(p-qA_1)\right)} = \frac{C_3}{C_2}\mathrm{e}^{-\pi\frac{\tau}{2}(E_1+E_2)}\frac{\left(m+E_2-(p-qA_2)\right)}{\left(m+E_1-(p-qA_1)\right)} \tag{155a}$$

and

$$b = \frac{G_{\mathrm{b}}}{G_{\mathrm{i}}} = \frac{\frac{1}{\sqrt{2}}\frac{C_4}{m}\mathrm{e}^{\pi\frac{\tau}{2}E_2}\left(m-E_2-(p-qA_2)\right)}{\frac{1}{\sqrt{2}}\frac{C_2}{m}\mathrm{e}^{\pi\frac{\tau}{2}E_1}\left(m+E_1-(p-qA_1)\right)} = \frac{C_4}{C_2}\mathrm{e}^{-\pi\frac{\tau}{2}(E_1-E_2)}\frac{\left(m-E_2-(p-qA_2)\right)}{\left(m+E_1-(p-qA_1)\right)}, \tag{155b}$$

which leads, using the approach in Sec. 3.2.4, to the probabilities

$$F = \frac{f^2}{b^2+f^2} \tag{156a}$$

and

$$B = \frac{b^2}{b^2+f^2}, \tag{156b}$$

where

$$\frac{C_3}{C_2} = \mathrm{e}^{\pi\frac{\tau}{2}(E_1+E_2)} \frac{[(2m-\mathscr{D}_1)\mathscr{F}_1+\mathscr{D}_2\mathscr{F}_2][(2m-\mathscr{D}_5)\mathscr{F}_5+\mathscr{D}_6\mathscr{F}_6]-[(2m+\mathscr{D}_1)\mathscr{F}_1-\mathscr{D}_2\mathscr{F}_2][(2m+\mathscr{D}_5)\mathscr{F}_5-\mathscr{D}_6\mathscr{F}_6]}{[(2m-\mathscr{D}_3)\mathscr{F}_3-\mathscr{D}_4\mathscr{F}_4][(2m-\mathscr{D}_5)\mathscr{F}_5+\mathscr{D}_6\mathscr{F}_6]-[(2m+\mathscr{D}_3)\mathscr{F}_3+\mathscr{D}_4\mathscr{F}_4][(2m+\mathscr{D}_5)\mathscr{F}_5-\mathscr{D}_6\mathscr{F}_6]} \tag{157a}$$

and

$$\frac{C_4}{C_2} = \mathrm{e}^{\pi\frac{\tau}{2}(E_1-E_2)} \frac{[(2m+\mathscr{D}_1)\mathscr{F}_1-\mathscr{D}_2\mathscr{F}_2][(2m-\mathscr{D}_3)\mathscr{F}_3-\mathscr{D}_4\mathscr{F}_4]-[(2m-\mathscr{D}_1)\mathscr{F}_1+\mathscr{D}_2\mathscr{F}_2][(2m+\mathscr{D}_3)\mathscr{F}_3+\mathscr{D}_4\mathscr{F}_4]}{[(2m-\mathscr{D}_3)\mathscr{F}_3-\mathscr{D}_4\mathscr{F}_4][(2m-\mathscr{D}_5)\mathscr{F}_5+\mathscr{D}_6\mathscr{F}_6]-[(2m+\mathscr{D}_3)\mathscr{F}_3+\mathscr{D}_4\mathscr{F}_4][(2m+\mathscr{D}_5)\mathscr{F}_5-\mathscr{D}_6\mathscr{F}_6]}, \tag{157b}$$

where

$$\mathscr{F}_1 = {}_2F_1(-\mu+\nu-\lambda, -\mu+\nu+\lambda; 1-2\mu; -1), \tag{158a}$$

$$\mathscr{F}_2 = {}_2F_1(-\mu+\nu-\lambda+1, -\mu+\nu+\lambda+1; 1-2\mu+1; -1), \tag{158b}$$

$$\mathscr{F}_3 = {}_2F_1(\lambda+\nu-\mu, \lambda+\nu+\mu; 1+2\lambda; -1), \tag{158c}$$

$$\mathscr{F}_4 = {}_2F_1(\lambda+\nu-\mu+1, \lambda+\nu+\mu+1; 1+2\lambda+1; -1), \tag{158d}$$

$$\mathscr{F}_5 = {}_2F_1(-\lambda+\nu-\mu, -\lambda+\nu+\mu; 1-2\lambda; -1), \tag{158e}$$

$$\mathscr{F}_6 = {}_2F_1(-\lambda+\nu-\mu+1, -\lambda+\nu+\mu+1; 1-2\lambda+1; -1) \tag{158f}$$

and

$$\mathscr{D}_1 = 2E_1 - (qA_2 - qA_1) - (p - qA_2) - (p - qA_1), \tag{159a}$$

$$\mathscr{D}_2 = i\frac{4}{\tau}\frac{(-\mu+\nu-\lambda)(-\mu+\nu+\lambda)}{1-2\mu}, \tag{159b}$$

$$\mathscr{D}_3 = 2E_2 + (qA_2 - qA_1) - (p - qA_2) - (p - qA_1), \tag{159c}$$

$$\mathscr{D}_4 = i\frac{4}{\tau}\frac{(\lambda+\nu-\mu)(\lambda+\nu+\mu)}{1-2\lambda}, \tag{159d}$$

$$\mathscr{D}_5 = 2E_2 - (qA_2 - qA_1) + (p - qA_2) + (p - qA_1), \tag{159e}$$

$$\mathscr{D}_6 = i\frac{4}{\tau}\frac{(-\lambda+\nu-\mu)(-\lambda+\nu+\mu)}{1-2\lambda}, \tag{159f}$$

with

$$\mu = i\sqrt{\alpha_1} = i\frac{\tau}{2}E_1, \tag{160a}$$

$$\nu = \frac{1-\sqrt{1-4(\alpha_0+\alpha_1+\alpha_2)}}{2} = i\frac{\tau}{2}(qA_2 - qA_1), \tag{160b}$$

$$\lambda = i\sqrt{\alpha_2} = i\frac{\tau}{2}E_2. \tag{160c}$$

In this analysis, E_1 and E_2 represent the incident electron energy E_i and the later-forward energy E_f, respectively, and the later-backward energy is defined as $E_\mathrm{b} = -E_\mathrm{f}$.

7.8 Dimensional Analysis for Time Constant τ

We have used natural units ($c = \hbar = 1$) for convenience throughout the calculations. However, to compare the theoretical results with experimental results, it is necessary to denormalize some of the obtained formulas. Specifically, we will need here to denormalize time in order to compare the transition times in the smooth potential with the de Broglie period of the electron.

This denormalization can be performed as follows. In *standard* units, which we shall distinguish from our natural units by tilde accents, the temporal part of the wavefunction takes the form[9, 13–17]

$$\psi \propto \mathrm{e}^{-i\tilde{E}\tilde{t}/\hbar}, \tag{161}$$

with $\tilde{E}$ and $\tilde{t}$ measured in joules and seconds, respectively. For simplicity, we express the energy, $\tilde{E}$, in terms of multiple of the electron rest mass energy, i.e., $\tilde{E} = \kappa\left(mc^2\right)$, which transforms Eq. (161) into

$$\psi \propto \mathrm{e}^{-i\kappa mc^2\tilde{t}/\hbar}. \tag{162}$$

Moreover, it makes sense to also express the *natural*-unit energy, E, in terms of that multiple (κ) of the electron mass, i.e., $E = \kappa m$, which alters Eq. (162) to

$$\psi \propto \mathrm{e}^{-iEc^2\tilde{t}/\hbar}. \tag{163}$$

Comparing this expression with its *natural*-unit counterpart,

$$\psi \propto \mathrm{e}^{-iEt}, \tag{164}$$

finally yields

$$t = \frac{c^2\tilde{t}}{\hbar}, \tag{165}$$

whose dimension is $1/m$ since the dimension of E is m.

Thus, the natural-unit dimensionless parameter τ in Eq. (100) denormalizes as

$$\tilde{\tau} = \tau\frac{\hbar}{mc^2}, \tag{166}$$

measured in seconds, which we call η in the paper.

8 Non-relativistic Regime

8.1 Non-relativistic Limit of the Dirac Equation

In order to determine the *non-relativistic limit*,

$$v \ll c, \tag{167}$$

of the Dirac equation, we shall first recast that equation in its (non-covariant) Schrödinger form because the covariant form, introduced in Sec. 2 and applied in the report to address the core of the problem, does not provide explicit access to the dynamic energy, which needs to be compared with the rest energy to determine that limit.

Let us start by recalling the covariant form of the Dirac equation, given by Eq. (8):

$$\left[\gamma^{\mu}(i\partial_{\mu} - qA_{\mu}) - m\right]\psi = 0. \tag{168}$$

Separating in this equation the temporal and spatial operators according to Einstein summation convention yields

$$\left[\gamma^{0}(i\partial_{0} - qA_{0}) + \gamma^{i}(i\partial_{i} - qA_{i}) - m\right]\psi = 0, \tag{169}$$

or, factoring out γ^0,

$$\gamma^0\left[i\partial_0 + \left(\gamma^0\right)^{-1}\gamma^i i\partial_i - qA_0 - \left(\gamma^0\right)^{-1}\gamma^i qA_i - \left(\gamma^0\right)^{-1}m\right]\psi = 0. \tag{170}$$

Dropping γ^0 and subsequently using Eq. (3) with the fact that, according to Eq. (1f), $\left(\gamma^0\right)^{-1} = \gamma^0$, simplifies that equation to

$$\left[i\partial_0 + \alpha^i i\partial_i - qA_0 - \alpha^i qA_i - \gamma^0 m\right]\psi = 0, \tag{171}$$

or, isolating the temporal derivative term,

$$i\partial_0\psi = \left[\alpha^i(-i\partial_i + qA_i) + \gamma^0 m + qA_0\right]\psi. \tag{172}$$

We may now write the last equation in vector form using the definitions

$$(\partial_0, \partial_i) \equiv \left(\frac{\partial}{\partial t}, \nabla\right), \tag{173a}$$

$$\alpha^i \equiv \boldsymbol{\alpha}, \tag{173b}$$

and

$$(A_0, A_i) \equiv (V, -\mathbf{A}). \tag{173c}$$

This yields

$$i\frac{\partial}{\partial t}\psi = \left[\boldsymbol{\alpha}\cdot(-i\nabla - q\mathbf{A}) + \gamma^0 m + qV\right]\psi, \tag{174}$$

which, using $\hat{\mathbf{p}} = -i\nabla$, becomes

$$i\frac{\partial}{\partial t}\psi = \mathscr{H}\psi, \tag{175a}$$

with the Hamiltonian

$$\mathscr{H} = \boldsymbol{\alpha}\cdot(\hat{\mathbf{p}} - q\mathbf{A}) + \gamma^0 m + qV, \tag{175b}$$

where

$$\boldsymbol{\alpha} = \begin{pmatrix} 0 & \boldsymbol{\sigma} \\ \boldsymbol{\sigma} & 0 \end{pmatrix}, \tag{175c}$$

with

$$\boldsymbol{\sigma} = (\sigma^1, \sigma^2, \sigma^3) \tag{175d}$$

being a vector whose components are the Pauli matrices given in Eq. (1d); specifically, it is a 1×3 vector of 2×2 matrices, so that α is a 4×4 matrix. Note that, in the above relations, the '$\cdot$' symbol represents the scalar product defined as $\mathbf{u} \cdot \mathbf{v} \triangleq \mathbf{u}\mathbf{v}^{\mathrm{T}}$, where T represents the transpose operation.

The Hamiltonian in Eq. (175b) has eigenvalues corresponding to the total relativistic energy of the particle,

$$E = E_{\mathrm{k}} + m + E_{\mathrm{p}}, \tag{176}$$

where E_{k}, m and

$$E_{\mathrm{p}} = qV \tag{177}$$

are the relativistic kinetic energy, rest mass energy, and potential energy, respectively. The wavefunction ψ solution to Eq. (175) for positive energies may then be expressed as

$$\psi = \bar{\psi}\mathrm{e}^{-imt} = \begin{pmatrix} \varphi \\ \vartheta \end{pmatrix} \mathrm{e}^{-imt}, \tag{178}$$

with separate the time evolution due to the rest mass, e^{-imt}, and kinetic and spatial energy dependencies, embedded in the modified wavefunction, $\bar{\psi}$. That separation will next allow us to get rid of the mass-related temporal dependence e^{-imt}. Indeed, inserting Eqs. (178), (175c) and (1f) into Eq. (175) yields

$$i\frac{\partial}{\partial t}\left\{\begin{pmatrix} \varphi \\ \vartheta \end{pmatrix} \mathrm{e}^{-imt}\right\} = \begin{pmatrix} \boldsymbol{\sigma}\cdot(\hat{\mathbf{p}} - q\mathbf{A})\vartheta \\ \boldsymbol{\sigma}\cdot(\hat{\mathbf{p}} - q\mathbf{A})\varphi \end{pmatrix} \mathrm{e}^{-imt} + m\begin{pmatrix} \varphi \\ -\vartheta \end{pmatrix} \mathrm{e}^{-imt} + qV\begin{pmatrix} \varphi \\ \vartheta \end{pmatrix} \mathrm{e}^{-imt}, \tag{179}$$

which, upon applying the product rule to the left-hand side derivative and multiplying the resulting equation by e^{imt} (and hence breaking covariance), simplifies to

$$i\frac{\partial}{\partial t}\begin{pmatrix} \varphi \\ \vartheta \end{pmatrix} = \begin{pmatrix} \boldsymbol{\sigma}\cdot(\hat{\mathbf{p}} - q\mathbf{A})\vartheta \\ \boldsymbol{\sigma}\cdot(\hat{\mathbf{p}} - q\mathbf{A})\varphi \end{pmatrix} - 2m\begin{pmatrix} 0 \\ \vartheta \end{pmatrix} + qV\begin{pmatrix} \varphi \\ \vartheta \end{pmatrix}. \tag{180}$$

The suppression of the exponential time evolution associated with the rest mass energy in this relation corresponds to a redefinition of the zero of energy that will not change the observable physics in the considered non-relativistic limit. In the non-relativistic regime, only energy differences, viz., $\Delta E = E_2 - E_1 = \hbar\omega$, with ω being the absorption or emission frequency, are observable. Changing the reference energy, such as by the rest-mass energy, E_{m}, does not change the observable, since $\Delta E = (E_2 + E_{\mathrm{m}}) - (E_1 + E_{\mathrm{m}}) = E_2 - E_1 = \hbar\omega$ represents the same (observable) absorption or emission frequency, whereas $E_{\mathrm{m}}t$ represents only a non-observable phase shift, $\Delta\phi = m$. This is different from the relativistic regime, where the rest-mass energy contributes to Lorentz invariance and can therefore not be simply subtracted. Another perspective is that no mass-to-energy conversion [$E_{\mathrm{k}} = mc^2(\gamma - 1)$ ($c = 1$ in natural units)] occurs in the non-relativistic limit, so the mass-related energy does not contribute to observable quantities in that limit. The observable physics will thus depend only on the kinetic and potential energies, associated with the modified wavefunction $\bar{\psi}$, and hence with φ and ϑ. Equation (180) is in fact a system of two coupled equations, which splits into

$$i\frac{\partial}{\partial t}\varphi = \boldsymbol{\sigma}\cdot(\hat{\mathbf{p}} - q\mathbf{A})\vartheta + E_{\mathrm{p}}\varphi \tag{181a}$$

and

$$i\frac{\partial}{\partial t}\vartheta = \boldsymbol{\sigma}\cdot(\hat{\mathbf{p}} - q\mathbf{A})\varphi - 2m\vartheta + E_{\mathrm{p}}\vartheta \tag{181b}$$

Let us now consider the non-relativistic limit. For this purpose, let us write the relativistic energy, in *standard units*, viz.,

$$E = \sqrt{(mc^2)^2 + (pc)^2} + E_{\mathrm{p}}, \tag{182a}$$

where

$$p = \frac{1}{\sqrt{1 - \frac{v^2}{c^2}}}mv. \tag{182b}$$

In the non-relativistic limit [Eq. (167)], $v \ll c$, the latter relation becomes

$$p \simeq mv, \tag{183a}$$

and inserting this new relation into Eq. (182a) yields

$$E = \sqrt{(mc^2)^2 + (mvc)^2} + E_\mathrm{p} = mc^2\sqrt{1 + \frac{v^2}{c^2}} + E_\mathrm{p}. \tag{183b}$$

Since $v \ll c$, the square root in the last expression may be approximated by its second-order Taylor expansion, which leads to

$$\begin{aligned} E &\simeq mc^2\left(1 + \frac{1}{2}\frac{v^2}{c^2}\right) + E_\mathrm{p} \\ &= mc^2 + \frac{1}{2}mv^2 + E_\mathrm{p} \\ &= mc^2 + \frac{p^2}{2m} + E_\mathrm{p} \\ &= mc^2 + E_\mathrm{k} + E_\mathrm{p}, \end{aligned} \tag{183c}$$

or, in natural units ($c = 1$),

$$E = m + E_\mathrm{k} + E_\mathrm{p}, \tag{184}$$

where (in that non-relativistic limit approximation) the rest energy term (m) is much larger than the (now simply $p^2/(2m)$) kinetic energy (E_k) and potential energy (E_p) contributions (see numerical example in Sec. 8.2).

With the above redefinition of the zero-energy level, the eigen-equation corresponding to the function ϑ in Eq. (181) may written

$$i\frac{\partial}{\partial t}\vartheta = W_\vartheta \vartheta, \tag{185a}$$

where, according to Eq. (184),

$$W_\vartheta = E - m = E_\mathrm{k} + E_\mathrm{p}, \tag{185b}$$

is the total mass-shifted energy, corresponding to the sum of the kinetic and potential energies. In the non-relativistic limit, since the kinetic and potential energies are negligible compared to the mass term, i.e.,

$$W_\vartheta \ll m, \tag{186}$$

so that, according to Eq. (185a),

$$i\frac{\partial}{\partial t}\vartheta \ll m, \tag{187}$$

we find, with $E_\mathrm{p} = qV \ll m$, that Eq. (181b) reduces to

$$\vartheta = \frac{\sigma \cdot (\hat{\mathbf{p}} - e\mathbf{A})}{2m}\varphi, \tag{188}$$

whose insertion into Eq. (181a) yields

$$i\frac{\partial}{\partial t}\varphi = \frac{1}{2m}[\sigma \cdot (\hat{\mathbf{p}} - q\mathbf{A})][\sigma \cdot (\hat{\mathbf{p}} - q\mathbf{A})]\varphi + qV\varphi. \tag{189}$$

The first term on the right-hand side of this equation may be written as $(\sigma \cdot \mathbf{a})(\sigma \cdot \mathbf{b})\varphi/(2m)$, where σ is the 1×3 vector of 2×2 matrices given by Eq. (175d) and $\mathbf{a} = \mathbf{b} = (\hat{\mathbf{p}} - q\mathbf{A})$ are 1×3 vectors. This expression may be decomposed into components with

$$(\sigma \cdot \mathbf{a})(\sigma \cdot \mathbf{b}) = (\sigma^i a^i)(\sigma^j b^j) = a^i b^j \sigma^i \sigma^j, \tag{190}$$

which is a 2×2 matrix, since a^i and b^j are scalar vector components and σ^i and σ^j are 2×2 matrix vector components [the Pauli matrices, given by Eq. (1d)]. The term $\sigma^i\sigma^j$ in the last equality can be written in an alternative, more convenient fashion, upon using the commutation and anticommuntation relations between the Pauli matrices[18], viz.,

$$\left[\sigma^i,\sigma^j\right]=\sigma^i\sigma^j-\sigma^j\sigma^i=2i\varepsilon^{ijk}\sigma^k, \tag{191a}$$

where ε^{ijk} is the Levi-Civita symbol, and

$$\left\{\sigma^i,\sigma^j\right\}=\sigma^i\sigma^j+\sigma^j\sigma^i=2\delta^{ij}, \tag{191b}$$

where δ^{ij} is the Kronecker delta, whose summation leads to

$$\sigma^i\sigma^j=i\varepsilon^{ijk}\sigma^k+\delta^{ij}. \tag{192}$$

Inserting Eq. (192) into Eq. (190) yields

$$\begin{aligned}(\sigma\cdot\mathbf{a})(\sigma\cdot\mathbf{b})&=a^ib^j\left(i\varepsilon^{ijk}\sigma^k+\delta^{ij}\right)\\&=i\varepsilon^{ijk}a^ib^j\sigma^k+a^ib^j\delta^{ij}\\&=i\underbrace{\varepsilon^{kij}a^ib^j}_{(\mathbf{a}\times\mathbf{b})^k}\sigma^k+a^ib^i\\&=i\sigma^k(\mathbf{a}\times\mathbf{b})^k+a^ib^i,\end{aligned} \tag{193}$$

which can alternatively be written, using again Einstein summation convention, in the vectorial form

$$(\sigma\cdot\mathbf{a})(\sigma\cdot\mathbf{b})=i\sigma\cdot(\mathbf{a}\times\mathbf{b})+\mathbf{a}\cdot\mathbf{b}. \tag{194}$$

Applying this result to the first term of the right-hand side of Eq. (189) yields

$$\begin{aligned}[\sigma\cdot(\hat{\mathbf{p}}-q\mathbf{A})][\sigma\cdot(\hat{\mathbf{p}}-q\mathbf{A})]&=i\sigma\cdot[(\hat{\mathbf{p}}-q\mathbf{A})\times(\hat{\mathbf{p}}-q\mathbf{A})]+(\hat{\mathbf{p}}-q\mathbf{A})\cdot(\hat{\mathbf{p}}-q\mathbf{A})\\&=i\sigma\cdot[\underbrace{(\hat{\mathbf{p}}\times\hat{\mathbf{p}})}_{=0}+q^2\underbrace{(\mathbf{A}\times\mathbf{A})}_{=0}-q(\hat{\mathbf{p}}\times\mathbf{A})-q(\mathbf{A}\times\hat{\mathbf{p}})]+(\hat{\mathbf{p}}-q\mathbf{A})^2\\&=i\sigma\cdot[-q(\hat{\mathbf{p}}\times\mathbf{A})-q(\mathbf{A}\times\hat{\mathbf{p}})]+(\hat{\mathbf{p}}-q\mathbf{A})^2\\&=-q\sigma\cdot[(\nabla\times\mathbf{A})+(\mathbf{A}\times\nabla)]+(\hat{\mathbf{p}}-q\mathbf{A})^2,\end{aligned} \tag{195}$$

where $\hat{\mathbf{p}}=-i\nabla$ has been used in the last equality. In the final expression, the term $[(\nabla\times\mathbf{A})+(\mathbf{A}\times\nabla)]$ is an operator and must therefore be evaluated conjointly with the wavefunction upon which it acts. Calling that wavefunction f, we find then

$$\begin{aligned}[(\nabla\times\mathbf{A}f)+(\mathbf{A}\times\nabla f)]^i&=\varepsilon^{ijk}\partial^jA^kf+\varepsilon^{ijk}A^j\partial^kf\\&=\varepsilon^{ijk}(\partial^jA^k)f+\varepsilon^{ijk}(\partial^jf)A^k+\varepsilon^{ijk}A^j(\partial^kf)\\&=\varepsilon^{ijk}(\partial^jA^k)f+\varepsilon^{ijk}(\partial^jf)A^k+\varepsilon^{ikj}A^k(\partial^jf)\\&=\varepsilon^{ijk}(\partial^jA^k)f+\cancel{\varepsilon^{ijk}(\partial^jf)A^k}-\cancel{\varepsilon^{ijk}(\partial^jf)A^k}\\&=\underbrace{\varepsilon^{ijk}(\partial^jA^k)}_{(\nabla\times\mathbf{A})^i}f,\end{aligned} \tag{196}$$

which reveals that

$$[(\nabla\times\mathbf{A}f)+(\mathbf{A}\times\nabla f)]=(\nabla\times\mathbf{A})f. \tag{197}$$

Setting in this relation $\nabla\times\mathbf{A}=\mathbf{B}$, where $\mathbf{B}$ is the magnetic field, according to Eq. (59b), and inserting the result into Eq. (195), we get

$$[\sigma\cdot(\hat{\mathbf{p}}-q\mathbf{A})][\sigma\cdot(\hat{\mathbf{p}}-q\mathbf{A})]=-q\sigma\cdot\mathbf{B}+(\hat{\mathbf{p}}-q\mathbf{A})^2. \tag{198}$$

Finally, substituting this identity into Eq (189) yields

$$i\frac{\partial}{\partial t}\varphi=\left[\frac{(\hat{\mathbf{p}}-q\mathbf{A})^2}{2m}-\frac{q}{2m}\sigma\cdot\mathbf{B}+qV\right]\varphi, \tag{199}$$

which is the *Schrödinger-Pauli* equation, where $q = -|q| = -e$ in the case of the electron.

In the absence of magnetic field ($\mathbf{B} = 0$), the term $\boldsymbol{\sigma} \cdot \mathbf{B}$ disappears, and Eq. (199) reduces to the ordinary Schrödinger equation,

$$i\frac{\partial}{\partial t}\varphi = \left[\frac{(\hat{\mathbf{p}} - q\mathbf{A})^2}{2m} + qV\right]\varphi. \tag{200}$$

The non-relativistic limit $v \ll c$ [Eq. (167)] is naturally valid in a large *range* of velocities ($\sim v < c/10$), which we hereafter refer to as the *non-relativistic regime*.

8.2 Numerical Example for the Relativistic Regime

Assuming standard units and the SI (Système International) system:

- electron rest mass: $m \simeq 9.109 \times 10^{-31}$ kg
- electron charge: $e \simeq 1.602 \times 10^{-19}$ C
- speed of light in vacuum: $c \simeq 2.998 \times 10^8$ m/s
- rest mass energy: $E_\mathrm{m} = mc^2 \simeq (9.109 \times 10^{-31})(2.998 \times 10^8)^2 \simeq 8.187 \times 10^{-14}$ J
- non-relativistic kinetic energy for $v = c/100$:
  $E_\mathrm{k} = \frac{1}{2}mv^2 = \frac{1}{2} \times (9.109 \times 10^{-31}) \times (2.998 \times 10^6)^2 \simeq 4.094 \times 10^{-18}$ J
- potential energy for $V = 7$ V (10 times the built-in potential of a silicon p-n junction):
  $E_\mathrm{p} = eV = (1.602 \times 10^{-19}) \times 7 \simeq 1.121 \times 10^{-18}$ J
- energy ratio: $\dfrac{E_\mathrm{k} + E_\mathrm{p}}{E_\mathrm{m}} = \dfrac{4.094 \times 10^{-18} + 1.121 \times 10^{-18}}{8.187 \times 10^{-14}} \simeq 6.370 \times 10^{-5} \simeq 0.00637\ \%$
- non-relativistic total energy with rest mass energy:
  $E_\mathrm{nr} = E_\mathrm{m} + E_\mathrm{k} + E_\mathrm{p} = 8.187 \times 10^{-14} + 4.094 \times 10^{-18} + 1.121 \times 10^{-18} \simeq 8.1875 \times 10^{-14}$
- relativistic total energy: $E_\mathrm{r} = \sqrt{(mc^2)^2 + (pc)^2} + qV$, where $p = \gamma mv$ with $\gamma = \frac{1}{\sqrt{1-\left(\frac{v}{c}\right)^2}} \simeq 1.00005$
  $\rightarrow p \simeq 2.731 \times 10^{-24}$ kg m/s
  $\Rightarrow E_\mathrm{r} = \sqrt{(9.109 \times 10^{-31})^2(2.998 \times 10^8)^4 + (2.731 \times 10^{-24})^2(2.998 \times 10^8)^2} + 1.121 \times 10^{-18}$
  $\simeq 8.1877 \times 10^{-14}\ J$
- error: $\dfrac{|E_\mathrm{r} - E_\mathrm{nr}|}{E_\mathrm{r}} = \dfrac{\left|8.1877 \times 10^{-14} - 8.1875 \times 10^{-14}\right|}{8.1877 \times 10^{-14}} \simeq 2.4427 \times 10^{-5} \triangleq 0.0024427\ \%$

8.3 Spatial Scattering Coefficients in the Non-relativistic Regime

For the sake of completeness, let us first consider the relativistic scattering coefficients for the scalar potential *spatial* step, which were given by Eq. (34) as

$$r = \frac{1 - \Gamma_\mathrm{s}}{1 + \Gamma_\mathrm{s}} \quad \text{and} \quad t = \frac{2}{1 + \Gamma_\mathrm{s}}, \tag{201a}$$

where

$$\Gamma_\mathrm{s} = \frac{(E - qV_2 - m)\sqrt{(E - qV_1)^2 - m^2}}{(E - qV_1 - m)\sqrt{(E - qV_2)^2 - m^2}}. \tag{201b}$$

Squaring Eq. (201b) and algebraically manipulating the resulting expression yields

$$
\begin{aligned}
\Gamma_{\mathrm{s}}^2 &= \frac{(E-qV_2-m)^2\left[(E-qV_1)^2-m^2\right]}{(E-qV_1-m)^2\left[(E-qV_2)^2-m^2\right]} \\
&= \frac{(E-qV_2-m)^2\left[(E-qV_1-m)(E-qV_1+m)\right]}{(E-qV_1-m)^2\left[(E-qV_2-m)(E-qV_2+m)\right]} \\
&= \frac{(E-qV_2-m)(E-qV_1+m)}{(E-qV_1-m)(E-qV_2+m)} \\
&= \frac{E^2-qV_1E-qV_2E+qV_1qV_2-mqV_2+mqV_1-m^2}{E^2-qV_2E-qV_1E+qV_1qV_2-mqV_1+mqV_2-m^2} \\
&= \frac{E^2-qV_1E-qV_2E+qV_1qV_2-mqV_2+mqV_1-m^2}{E^2-qV_2E-qV_1E+qV_1qV_2-mqV_1+mqV_2-m^2}-1+1 \\
&= 1-\frac{2m(qV_2-qV_1)}{E^2-qV_2E-qV_1E+qV_1qV_2-mqV_1+mqV_2-m^2} \\
&= 1-\frac{2m(qV_2-qV_1)}{(E-qV_1)^2-m^2-(qV_1)^2+qV_1E-qV_2E+qV_1qV_2+m(qV_2-qV_1)} \\
&= 1-\frac{2m(qV_2-qV_1)}{(E-V_1)^2-m^2-(qV_2-qV_1)(E-qV_1-m)} \\
&= 1-\frac{2m(qV_2-qV_1)}{[(E-qV_1)^2-m^2]\left[1-(qV_2-qV_1)\dfrac{E-qV_1-m}{(E-qV_1)^2-m^2}\right]} \\
&= 1-\frac{(qV_2-qV_1)}{\dfrac{(E-qV_1)^2-m^2}{2m}\left[1-(qV_2-qV_1)\dfrac{E-qV_1-m}{(E-qV_1)^2-m^2}\right]} \\
&= 1-\frac{(qV_2-qV_1)}{\dfrac{p_{\mathrm{i}}^2}{2m}\left[1-(qV_2-qV_1)\dfrac{1}{E-qV_1+m}\right]}.
\end{aligned}
\tag{202}
$$

In the non-relativistic regime [Eq. (186)],

$$E_{(\mathrm{r})}-\cancel{qV_1}+m>>qV_2-\cancel{qV_1}, \tag{203}$$

and the kinetic energy $p_{\mathrm{i}}^2/2m$ is simply

$$\frac{p_{\mathrm{i}}^2}{2m}=E_{(\mathrm{nr})}-qV_1, \tag{204}$$

where $E_{(\mathrm{nr})}$ is non-relativistic total energy.

Inserting Eqs. (203) and (204) into Eq. (202) yields then

$$\Gamma_{\mathrm{s}}^2\simeq 1-\frac{qV_2-qV_1}{E-qV_1} \tag{205}$$

and

$$\Gamma_{\mathrm{s}}\simeq\sqrt{1-\frac{qV_2-qV_1}{E-qV_1}}=\sqrt{\frac{E-qV_2}{E-qV_1}}=\sqrt{\frac{2m(E-qV_2)}{2m(E-qV_1)}}. \tag{206}$$

Since the non-relativistic total energy is $E_{(\mathrm{nr})}=p^2/2m+qV=\hbar^2k^2/2m+qV$, where $\hbar=1$ in natural units,

$$\sqrt{2m(E-qV_{1,2})}=k_{1,2}, \tag{207}$$

where $k_{1,2}$ are wave numbers, so that Eq. (206) reduces to

$$\Gamma_{\mathrm{s}} \simeq \frac{k_2}{k_1}. \tag{208}$$

Inserting Eq. (208) into Eq. (201a) leads to the non-relativistic reduction of the Dirac reflection and transmission coefficients to the Schrödinger scattering coefficients

$$r = \frac{1-\dfrac{k_2}{k_1}}{1+\dfrac{k_2}{k_1}} = \frac{k_1-k_2}{k_1+k_2} \quad \text{and} \quad t = \frac{2}{1+\dfrac{k_2}{k_1}} = \frac{2k_1}{k_1+k_2}. \tag{209}$$

Note that the corresponding solution to the Schrödinger equation [Eq. (200)] is here [for the potential given in (29)] readily found as

$$\psi_1 = \mathrm{e}^{-iE_{\mathrm{i}}t}\mathrm{e}^{ip_{\mathrm{i}}z} + r\mathrm{e}^{-iE_{\mathrm{r}}t}\mathrm{e}^{ip_{\mathrm{r}}z} \tag{210a}$$

and

$$\psi_2 = t\mathrm{e}^{-iE_{\mathrm{t}}t}\mathrm{e}^{ip_{\mathrm{t}}z}, \tag{210b}$$

where

$$E_{\mathrm{i}} = E_{\mathrm{r}} = E_{\mathrm{t}} = E, \quad p_{\mathrm{i}} = \sqrt{2m(E-qV_1)}, \quad p_{\mathrm{r}} = -p_{\mathrm{i}} \quad \text{and} \quad p_{\mathrm{t}} = \sqrt{2m(E-qV_2)}. \tag{210c}$$

which implies the same scattering coefficients r and t as in Eq. (209).

8.4 Temporal Scattering Coefficients in the Non-relativistic Regime

The relativistic scattering coefficients for the vector potential temporal step were given by Eq. (53) as

$$f = \frac{1+\Gamma_{\mathrm{t}}}{2\Gamma_{\mathrm{t}}} \quad \text{and} \quad b = \frac{\Gamma_{\mathrm{t}}-1}{2\Gamma_{\mathrm{t}}}, \tag{211a}$$

with

$$\Gamma_{\mathrm{t}} = \frac{\dfrac{E_f}{p-qA_2}}{\dfrac{E_{\mathrm{i}}-m}{p-qA_1} + \dfrac{m}{p-qA_2}} = \frac{\dfrac{\sqrt{(p-qA_2)^2+m^2}}{p-qA_2}}{\dfrac{\sqrt{(p-qA_1)^2+m^2}-m}{p-qA_1} + \dfrac{m}{p-qA_2}}, \tag{211b}$$

where Eqs. (50b) and (50c) has been used in the last relation.

Squaring Eq. (211b) and algebraically manipulating the resulting expression yields

$$\begin{aligned}
\Gamma_{\mathrm{t}}^2 &= \frac{(p-qA_2)^2+m^2}{\left[\dfrac{p-qA_2}{p-qA_1}\left(\sqrt{(p-qA_1)^2+m^2}-m\right)+m\right]^2} \\
&= \frac{(p-qA_2)^2+m^2}{\left(\dfrac{p-qA_2}{p-qA_1}\right)^2\left(\sqrt{(p-qA_1)^2+m^2}-m\right)^2 + 2\dfrac{p-qA_2}{p-qA_1}\left(\sqrt{(p-qA_1)^2+m^2}-m\right)m+m^2} \\
&= \frac{\left(\dfrac{p-qA_2}{m}\right)^2+1}{\left(\dfrac{p-qA_2}{p-qA_1}\right)^2\left(\sqrt{\left(\dfrac{p-qA_1}{m}\right)^2+1}-1\right)^2 + 2\dfrac{p-qA_2}{p-qA_1}\left(\sqrt{\left(\dfrac{p-qA_1}{m}\right)^2+1}-1\right)+1}.
\end{aligned} \tag{212}$$

In the non-relativistic regime [Eq. (186)],

$$m(c^2) \gg p(c) - qA_{1,2} = (c)\sqrt{2mE_\mathrm{k}}, \quad \text{(standard units)} \tag{213}$$

and the square roots in the last expression may therefore be approximated by their second-order Taylor expansion, which leads to

$$\begin{aligned}
\Gamma_\mathrm{t}^2 &\simeq \frac{\left(\frac{p-qA_2}{m}\right)^2+1}{\left(\frac{p-qA_2}{p-qA_1}\right)^2\left(\frac{1}{2}\left(\frac{p-qA_1}{m}\right)^2+1-1\right)^2+2\frac{p-qA_2}{p-qA_1}\left(\frac{1}{2}\left(\frac{p-qA_1}{m}\right)^2+1-1\right)+1} \\
&= \frac{\left(\frac{p-qA_2}{m}\right)^2+1}{\left(\frac{p-qA_2}{p-qA_1}\right)^2\left(\frac{1}{2}\left(\frac{p-qA_1}{m}\right)^2\right)^2+2\frac{p-qA_2}{p-qA_1}\left(\frac{1}{2}\left(\frac{p-qA_1}{m}\right)^2\right)+1} \\
&= \frac{\left(\frac{p-qA_2}{m}\right)^2+1}{\frac{1}{4}\left(\frac{p-qA_1}{m}\right)^2\left(\frac{p-qA_2}{m}\right)^2+\left(\frac{p-qA_1}{m}\right)\left(\frac{p-qA_2}{m}\right)+1} \\
&= \frac{\left(\frac{p-qA_2}{m}\right)^2+1}{\left(\frac{1}{2}\left(\frac{p-qA_1}{m}\right)\left(\frac{p-qA_2}{m}\right)+1\right)^2}
\end{aligned} \tag{214}$$

and

$$\begin{aligned}
\Gamma_\mathrm{t} &= \frac{\sqrt{\left(\frac{p-qA_2}{m}\right)^2+1}}{\frac{1}{2}\left(\frac{p-qA_1}{m}\right)\left(\frac{p-qA_2}{m}\right)+1} \\
&\simeq \frac{\frac{1}{2}\left(\frac{p-qA_2}{m}\right)^2+1}{\frac{1}{2}\left(\frac{p-qA_1}{m}\right)\left(\frac{p-qA_2}{m}\right)+1}.
\end{aligned} \tag{215}$$

Since $m(c^2) \gg p(c) - qA_{1,2}$, the terms $[(p-qA_{1,2})/m]^2$ are negligible. Therefore,

$$\Gamma_\mathrm{t} \simeq 1. \tag{216}$$

Inserting Eq. (216) into Eq. (211a) leads to the non-relativistic reduction of the Dirac reflection and transmission coefficients

$$f = 1 \quad \text{and} \quad b = 0, \tag{217}$$

which are the same as those obtained from the Schrödinger equation.

Note that the corresponding solution to the Schrödinger equation [Eq. (200)] is here [for the potential given in (48)] readily found as

$$\psi_1 = \mathrm{e}^{-iE_\mathrm{i}t}\mathrm{e}^{ip_\mathrm{i}z} \tag{218a}$$

and

$$\psi_2 = f\mathrm{e}^{-iE_\mathrm{f}t}\mathrm{e}^{ip_\mathrm{f}z}, \tag{218b}$$

where

$$p_{\mathrm{i}} = p_{\mathrm{f}} = p, \quad E_{\mathrm{i}} = \frac{(p - qA_1)^2}{2m} \quad \text{and} \quad E_{\mathrm{f}} = \frac{(p - qA_2)^2}{2m}. \tag{218c}$$

which implies the later-forward coefficient $f = 1$ and later-backward coefficient $b = 0$, consistently with Eq. (217).

References

1. Dirac, P. A. M. The quantum theory of the electron. *Proc. R. Soc. Lond. A* **117**, 610–624 (1928).
2. Greiner, W. *Relativistic Quantum Mechanics. Wave Equations* (Springer, Berlin, 2000), 3rd ed edn.
3. Jackson, J. D. *Classical Electrodynamics* (Wiley, 1998), 3rd ed edn.
4. Peskin, M. & Schroeder, D. *An Introduction to Quantum Field Theory* (CRC press, 2018).
5. Morgenthaler, F. R. Velocity Modulation of Electromagnetic Waves. *IEEE Trans. Microw. Theory Tech.* **6**, 167–172, DOI: 10.1109/TMTT.1958.1124533 (1958).
6. Caloz, C. & Deck-Léger, Z.-L. Spacetime metamaterials, part II: Theory and applications. *IEEE Trans. Antennas Propag.* **68**, 1583–1598, DOI: 10.1109/TAP.2019.2944216 (2020).
7. Noether, E. Invariante Variationsprobleme. *Nach. Ges. Wiss. Gött.* **1918**, 235–257 (1918).
8. Jackson, J. D. & Okun, L. B. Historical roots of gauge invariance. *Rev. Mod. Phys.* **73**, 663–680, DOI: 10.1103/RevModPhys.73.663 (2001).
9. Griffiths, D. J. & Schroeter, D. F. *Introduction to Quantum Mechanics* (Cambridge University Press, 2018), 3 edn.
10. Caloz, C. & Deck-Léger, Z.-L. Spacetime metamaterials, part I: General concepts. *IEEE Trans. Antennas Propag.* **68**, 1569–1582, DOI: 10.1109/TAP.2019.2944225 (2020).
11. Gradshteyn, I. S. & Ryzhik, I. M. *Table of Integrals, Series, and Products* (Academic Press, 2014).
12. Das, A. *Lectures On Quantum Field Theory (Second Edition)* (World Scientific Publishing Company, 2020).
13. Zettili, N. *Quantum Mechanics: Concepts and Applications* (Wiley, 2009).
14. Shankar, R. *Principles of Quantum Mechanics* (Springer US, 2012).
15. Miller, D. A. *Quantum Mechanics for Scientists and Engineers* (Cambridge University Press, 2008).
16. Landau, L. & Lifshitz, E. *Quantum Mechanics: A Shorter Course of Theoretical Physics* (Elsevier Science, 2013).
17. Sakurai, J. J. & Napolitano, J. *Modern Quantum Mechanics* (Cambridge University Press, 2020), 3 edn.
18. Ryder, L. H. *Quantum Field Theory* (Cambridge University Press, 1996).
